# Non-destructive collection and metabarcoding of arthropod environmental DNA remained on a terrestrial plant

**DOI:** 10.1038/s41598-023-32862-4

**Published:** 2023-05-12

**Authors:** Kinuyo Yoneya, Masayuki Ushio, Takeshi Miki

**Affiliations:** 1 Faculty of Agriculture, Kindai University, 3327-204, Nakamachi, Nara 631-8505 Japan; 2 Center for Biodiversity Science, Ryukoku University, 1-5 Yokotani, Seta Oe-cho, Otsu, Shiga 520-2194 Japan; 3 Hakubi Center, Kyoto University, Kyoto, 606-8501 Japan; 4 Center for Ecological Research, Kyoto University, Otsu, 520-2113 Japan; 5 Department of Ocean Science, The Hong Kong University of Science and Technology, Clear Water Bay, Kowloon, Hong Kong SAR China; 6 Faculty of Advanced Science and Technology, Ryukoku University, 1-5 Yokotani, Seta Oe-cho, Otsu, Shiga 520-2194 Japan

**Keywords:** Ecology, Biodiversity

## Abstract

Reliable survey of arthropods is a crucial for their conservation, community ecology, and pest control on terrestrial plants. However, efficient and comprehensive surveys are hindered by challenges in collecting arthropods and identifying especially small species. To address this issue, we developed a non-destructive environmental DNA (eDNA) collection method termed “plant flow collection” to apply eDNA metabarcoding to terrestrial arthropods. This involves spraying distilled or tap water, or using rainfall, which eventually flows over the surface of the plant, and is collected in a container that is set at the plant base. DNA is extracted from collected water and a DNA barcode region of cytochrome c oxidase subunit I (COI) gene is amplified and sequenced using a high-throughput Illumina Miseq platform. We identified more than 64 taxonomic groups of arthropods at the family level, of which 7 were visually observed or artificially introduced species, whereas the other 57 groups of arthropods, including 22 species, were not observed in the visual survey. These results show that the developed method is possible to detect the arthropod eDNA remained on plants although our sample size was small and the sequence size was unevenly distributed among the three water types tested.

## Introduction

Arthropod species in terrestrial ecosystems are diverse^1^. The diversity of arthropods is in particular influenced by the diversity of plants that the arthropods interact with^2,3^. The survey of various arthropods is a crucial step in studies of the conservation and community ecology of arthropods on plants and pest management in agriculture. Researchers have invested considerable efforts in identifying and exploring arthropods. However, arthropods are frequently very small and occasionally exhibit limited morphological variation. Unfortunately, the number of taxonomists that can identify or describe arthropods species based on morphology is decreasing^4^. In addition, specimen-based surveys and complete arthropod community assessments are often time-consuming and costly^5^ because of the hiding behavior, concealed coloration, and relatively high mobility of many specimens. Therefore, assessing and distinguishing arthropods is difficult, which in turn prevents the very much needed rapid and accurate surveys.

DNA metabarcoding, which combines PCR using universal primers and high-throughput sequencing, enables species diversity surveys based on genetic similarity and is a promising tool to overcome the difficulties in species identification. Fragments of the cytochrome c oxidase I (COI) region of the mitochondrial DNA have been frequently used for species identification of arthropods^6,7^. However, the method also requires collecting arthropods with traps, nets, or other methods. Environmental DNA (eDNA) metabarcoding has recently gained attention as a tool for efficient biodiversity monitoring, particularly in aquatic systems^8^. eDNA is genetic material originating from organisms, such as metabolic waste and tissues from the body surface^9,10^. It has been shown to be useful for investigating species that are difficult to find because of low population density or behavioral traits (e.g., nocturnal and quick-moving behavior)^11,12^. eDNA metabarcoding enables us to estimate species richness in a certain water area^13^. Thus, eDNA metabarcoding could contribute to ecological research and biota monitoring on large scale and in the long term^10,13^. eDNA metabarcoding has also been applied in terrestrial ecosystems to detect mammals living in forest ecosystems^14^, pollinators^15,16^, and local arthropod diversity^17,18^. However, eDNA metabarcoding has been rarely applied to organisms associated with terrestrial plants except for various pollinator species from flowers^16^ and invertebrates from tree canopies^19^ because methods to collect eDNA remaining on plant surfaces are still developing best practice advises. In several cases, the eDNA of herbivores remaining on plants has been successfully extracted using destructive methods^20–23^. For example, ungulate browsing preference was investigated by analyzing animal eDNA in saliva that adhered to a bite site on twigs^21,22^.

A method to collect eDNA from plants, such as crops and endangered or protected plants, without damaging them would be preferred. Several studies have proposed potentially noninvasive methods for collecting eDNA from terrestrial plants. For example, to detect an invasive spotted lanternfly, *Lycorma delicatula,* eDNA was collected using a cotton roller and sprayed with water on a part of a plant^24^.

In the present study, we investigated a non-destructive method for the eDNA metabarcoding of arthropod communities on a plant. Our targets included herbivores feeding, temporally visiting, or staying on a plant and predators possibly feeding on preys on a plant. As in other studies, we focused on the utility of water as a collection medium for terrestrial eDNA^23,24^. To this end, we sprayed distilled or tap water on the whole body of an eggplant and cabbage growing in a pot or on the ground in a field and collected the water flow at the base of plants, assuming that the water running through the whole plant body contains arthropod eDNA remaining on the plant. In order to disseminate this technique to detect pest insect attacks and apply it to large-scale biodistribution surveys (e.g., with citizen participants), we primarily aimed to evaluate a method of collecting eDNA using tap water, which is more convenient to use than distilled water in Japan. In addition, we investigated the effectiveness of collecting eDNA using rainfall, which has been suggested as a promising alternative in fields^19^ as it can eliminate the need for manual water addition during the collection process. However, it should be noted that our study was not intended to identify the optimal water type for eDNA collection, as our sample size was small and the sequence size was unevenly distributed among the three water types (tap water, distilled water, and rainfall).

## Results and discussions

### DNA metabarcoding reads

Sequencing of the 44 libraries, together with other 12 libraries (total number of libraries = 56), yielded a total of 1,201,786 raw reads, with an average of 94.29% base calls being quality scores of more than 30.00. After primer trimming, quality filtering, merging of paired reads, and chimera filtering, a total of 923,828 reads for the focal 44 libraries (mean ± s.e.: 20,996 ±  8506 reads per library, Table S1) were clustered into 1,512 ASVs (Table S2). 792.7 ± 714.6 (mean ±  s.e.) reads per library were detected in the PCR and field-negative controls and were clustered into 45 ASVs, which included 11 Arthropoda ASVs. The most abundant sequence among these 45 ASVs was a sequence of Diptera (total 3086 reads). However, this ASV was only occurred in the PCR-negative controls (n = 2) but not in the other 42 samples. Thus, this ASV was possibly regent contamination. Each of the other 10 Arthropoda ASVs in the negative controls included less than five reads respectively. Of the other 34 ASVs, 16 ASVs could not be assigned until kingdom level. Seven, six, four, and one ASVs were assigned as Discosea, Fungi, and non-Arthropoda Metazoa including *Homo sapience,* Gastrotricha, and Rotifera, and Chrysopyceae, respectively. Two PCR- (S55, S56) and five field- (S-44-S48) negative controls included eight and four Arthropoda ASVs, respectively. Only one of them (Tetranychus) from the PCR-negative controls was detected as a single read in one of the five field-negative controls (S44-S48). The other three Arthropoda ASVs from the field-negative controls were not shared with the PCR-negative controls; one read each of unidentified Insecta ASV, Insecta (*Thrips Palmi*) ASV, and Collembola ASV were detected in different samples. These also included non-Arthropoda ASVs, but most of them had one or two reads from one or two samples only. An unidentified ASV at the Kingdom level was detected in all five field-negative controls. The maximum read count for this ASV in a single filed negative control was 142, and it was also detected in most of the other 39 samples, suggesting a common source of contamination that likely affected the PCR-negative controls as well as many of the other samples. The results suggest that the total sampling volume of 18 L (for 3-h sampling) to 288 L (for 48-h sampling) of air per sample were not sufficient for collecting aerial eDNA, especially considering that the sampling volumes used in the previous study were much larger (6000–9000 L^18^). Additionally, the abundance of aerial eDNA in the glass house was too low to be detected even with a greater sampling volume. At the same time, it demonstrates that the contamination levels during the DNA extraction and PCR steps were low. It should be also noted that we could not distinguish contaminations of aerial eDNA and those that occurred during the extraction steps because the extraction negative controls were not prepared. After subtraction of the sum of reads from each of ASVs that were present in the negative controls, a total of 914,710 and 24,722 ± 10,011 (mean ± s.e.) reads per library remained for further analyses (when the subtraction generated the negative read numbers, these were converted into zero). Among 173 ASVs assigned to Arthropoda, 54, 31, and 17 ASVs were identified up to species, genus and family levels, respectively (Table S2).

Across all samples, 64 taxonomic groups of arthropods, which were assigned at least to the family level, were detected, belonging to 15 orders, 42 families, 46 genera, and 29 species (Tables S3 and 1). Of these, seven were *Aphis gossypii, Pieris rapae, Plutella xylostella, Myzus persicae, Brevicoryne brassicae,* a leaf miner fly, *Liriomyza sativae* and *Sphaerophoria macrogaster*, which were artificially introduced or visually observed on experimental plants. They were hereafter termed “target species” in this study. An additional 57 arthropods were neither introduced nor observed by visual survey.

**Table 1:** List of taxonomic information of arthropods identified in eDNA samples collected from plant surface using the “plant flow collection” method (see, Fig. 2).

Common group name	Class	Order	Family	Genus		Species
Spider	Arachnida	Araneae	Tetragnathidae	*Leucauge*		*–*
Predatory mite	Arachnida	Mesostigmata	Phytoseiidae	*Euseius*		*–*
Mite	Arachnida	Sarcoptiformes	Acaridae	*Tyrophagus*		*Tyrophagus putrescentiae*
Mite	Arachnida	Trombidiformes	Eriophyidae	*–*		*–*
Mite	Arachnida	Trombidiformes	Eupodidae	*–*		*–*
Mite	Arachnida	Trombidiformes	Tarsonemidae	*–*		*–*
Mite	Arachnida	Trombidiformes	Tenuipalpidae	*Brevipalpus*		*–*
Mite	Arachnida	Trombidiformes	Tetranychidae	*Tetranychus*		–
Springtail	Collembola	Entomobryomorpha	Entomobryidae	*Entomobrya*		–
Springtail	Collembola	Entomobryomorpha	Isotomidae	*Desoria*		–
Springtail	Collembola	Symphypleona	Bourletiellidae	–		–
Springtail	Collembola	Symphypleona	Katiannidae	*Sminthurinus*		–
Beetle	Insecta	Coleoptera	Carabidae	*Harpalus*		*–*
Beetle	Insecta	Coleoptera	Corylophidae	*Pheropsophus*		*Pheropsophus jessoensis*
Beetle	Insecta	Coleoptera	Corylophidae	*Sericoderus*		*Sericoderus lateralis*
Fly	Insecta	Diptera	Agromyzidae	*Liriomyza*		*Liriomyza sativae*
Fly	Insecta	Diptera	Cecidomyiidae	*–*		*––*
Fly	Insecta	Diptera	Chironomidae	*Cladotanytarsus*		*Cladotanytarsus vanderwulpi*
Fly	Insecta	Diptera	Culicidae	*Aedes*		*Aedes albopictus*
Fly	Insecta	Diptera	Drosophilidae	*Drosophila*		*–*
Fly	Insecta	Diptera	Drosophilidae	*Scaptomyza*		*Scaptomyza pallida*
Fly	Insecta	Diptera	Psychodidae	*Psychoda*		
Fly	Insecta	Diptera	Syrphidae	*Sphaerophoria*		*Sphaerophoria macrogaster*
Fly	Insecta	Hemiptera	Aleyrodidae	*Trialeurodes*		*Trialeurodes vaporariorum*
Aphid	Insecta	Hemiptera	Aphididae	*Aphis*		*Aphis gossypii*
Aphid	Insecta	Hemiptera	Aphididae	*Brevicoryne*		*Brevicoryne brassicae*
Aphid	Insecta	Hemiptera	Aphididae	*Myzus*		*Myzus persicae*
Bug	Insecta	Hemiptera	Miridae	Creontiades		Creontiades coloripes
Aphid	Insecta	Hemiptera	Pemphigidae	*Tetraneura*		*–*
Bug	Insecta	Hemiptera	Pentatomidae	*Eurydema*		*Eurydema gebleri*
Bug	Insecta	Hemiptera	Pseudococcidae	*Planococcus*		*Planococcus citri*
Parasitoid wasp	Insecta	Hymenoptera	Braconidae	*Aphidius*		*Aphidius colemani*
Parasitoid wasp	Insecta	Hymenoptera	Braconidae	*Cotesia*		*Cotesia vestalis*
Parasitoid wasp	Insecta	Hymenoptera	Braconidae	*Diaeretiella*		*Diaeretiella rapae*
Parasitoid wasp	Insecta	Hymenoptera	Eulophidae	Neochrysocharis		
Parasitoid wasp	Insecta	Hymenoptera	Figitidae	*Kleidotoma*		*–*
Parasitoid wasp	Insecta	Hymenoptera	Pteromalidae	*–*		*–*
Parasitoid wasp	Insecta	Hymenoptera	Trichogrammatidae	*Trichogramma*		*–*
Moth	Insecta	Lepidoptera	Crambidae	*Bradina*		*Bradina diagonalis*
Moth	Insecta	Lepidoptera	Crambidae	*Udea*		*–*
Moth	Insecta	Lepidoptera	Noctuidae	*Autographa*		*–*
Moth	Insecta	Lepidoptera	Noctuidae	*Mamestra*		*Mamestra brassicae*
Butterfly	Insecta	Lepidoptera	Pieridae	*Pieris*		*Pieris rapae*
Moth	Insecta	Lepidoptera	Plutellidae	*Plutella*		*Plutella xylostella*
Moth	Insecta	Lepidoptera	Pyralidae	Ephestia		Ephestia kuehniella
Mantis	Insecta	Mantodea	Mantidae	*Tenodera*		*Tenodera sinensis*
Grasshoppers	Insecta	Orthoptera	Tetrigidae	*Tetrix*		*Tetrix japonica*
Booklice	Insecta	Psocoptera	Liposcelidae	*Liposcelis*		*–*
Booklice	Insecta	Psocoptera	Trogiidae	*Cerobasis*		*Cerobasis guestfalica*
Thrip	Insecta	Thysanoptera	Thripidae	*Frankliniella*		*Frankliniella occidentalis*
Thrip	Insecta	Thysanoptera	Thripidae	*Scirtothrips*		*Scirtothrips dorsalis*
Thrip	Insecta	Thysanoptera	Thripidae	*Thrips*		*Thrips tabaci*

### eDNA collected from potted eggplant

Cotton aphids *A. gossypii* were detected in all eDNA samples collected from eggplants using tap water (4/4 samples, Tables 2 and 3) and distilled water (2/2 samples, in Tables 2 and 3). Thus, both tap water and distilled water appeared to be effective media for eDNA collection (Table 3). It is highly recommended to filter water samples as soon as possible after collecting eDNA using tap water because residual chloride in tap water can degrade the collected eDNA.

**Table 2:** An overview of sample information and detection rate of eDNA from target species per sampling condition such as type of water, feeding condition, target species, and season.

Plant (N*^1^)	Pot or field	Water type*^2^	Target species*^4^	Month	Detection ratio	Total samples	Details in
Eggplant (14)	Pot	TW	*A. gossypii* (leafminer)	Jun	3/3 (3/3)	4	Table 3 (Fig S1)
				Jul	1/1 (1/1)		
		TW	leafminer	Aug	1/4	8	Fig S1
				Sep	1/4		
		DW	*A. gossypii*	Oct	2/2	2	Table 3 (Fig S1)
Cabbage (24)	Pot	TW	*B. brassicae*	Jun	0/2	6	Table 5
				July	4/4		
			*M. persicae*	Jun	1/1	3	
				July	2/2		
			*P. rapae*	Jun	1/2	6	Table 6
				July	3/4		
			*P. xylostella*	July	1/2	2*^5^	
			chewing herbivore	Jun	1/1	3	Table S4
				July	2/2		
	Field	TW	*B. brassicae*	July	1/1	2	Figure 1
			*M. persicae*		0/1		
			*P. rapae*		2/2		
		Rainfall	*P. rapae*	July	3/3	3	Figure 1
			*P. xylostella*		3/3		
			Total		38/50	37	
			(excl. samples of leaf miner and chewing herbivore)*^6^		(29/35)	(26)	

*^1^The number of samples.

*^2^TW: tap water, DW: distilled water.

*^3^Presence or absence (feeding track was presence) of feeding species on sampling plants.

*^4^Target species were observed or artificially introduced into a plant. Leaf miners and chewing herbivores were not observed directly but there were feeding track remained on plants.

*^5^The two samples collected eDNA from plants that *P. xylostella* was visually observed on, were the same plants that *P. rapae* was feeding on in July.

*^6^Total of all samples excluding samples that eDNA was collected from a plant with feeding track but without direct observations of the herbivores.

**Table 3:** The number of *Aphis gossypii* on a potted eggplant at the sampling day and total sequence reads detected from tap water or distilled water samples collected from plant surface by the “plant flow collection” method.

Sample ID	No. of aphids	Water type*^3^	Sequence reads
S1	39	TW	143
S2	50	TW	38
S3	157	TW	179
S27	5	TW	22
S49*^1^	Few C*^2^	DW	305
S50	Few C*^2^	DW	19

The amount of water and sampling day was described in Table 3.

*^1^Three egg plants were used for sampling.

*^2^The number of aphids was not counted, but we chose plants with few colonies (C).

*^3^Tap water (TW) or distilled water (DW) was used to collect eDNA. The water volumes and collection days are listed in Table 3.

Most eggplants were infested by leaf miners in our greenhouse this year, and many feeding tracks remained on the leaves. Normally leaf miners occurred throughout the year in greenhouses in Japan. In June and July, three and one potted eggplants inoculated with *A.gossypii* had leaf miner tracks, respectively. eDNA of a vegetable leaf miner, *L*. *sativae*, was detected from all of these eggplants. Among four eggplants that had leaf miner tracks but without *A.gossypii* in August and September respectively, eDNA of *L. sativae* was detected on one eggplant in each month (Tables 2 and 4, Fig. S1). eDNA of *L. sativae* was not detected in October from two eggplants infested by aphids but not by leaf miners. In total, eDNA of *L. sativae* was detected on six eggplants among 12 eggplants that had leaf miner tracks (Table 2). In June and July, when *L.sativae* was detected in all samples, *L.sativae* may have been engaged in feeding behavior on plants. On the other hand, in August and September, there were still traces of feeding damage, but *L. sativae* may have left most of the plants and eDNA may have already been degraded or washed away when watering plants (e.g., see Valentin et al*.*^25^).

**Table 4:** List of sample information.

Sample ID	Condition of plant	Plant	Type of water*^2^	Sprayed water volume (ml)	Filtered water volume (ml)	Sampling day	Target species*^3^	Number of herbivores*^7^	Developmental stage
S01	Pot	Eggplant	TW	250	90	2017/6/27	*A. gossypii*	39	–
S02	Pot	Eggplant	TW	250	80	2017/6/27	*A. gossypii*	50	–
S03	Pot	Eggplant	TW	250	100	2017/6/27	*A. gossypii*	157	–
S27	Pot	Eggplant	TW	250	100	2017/7/14	A. gossypii*^4^	5	–
S49	Pot	Eggplant*^1^	DW	500	350	2017/10/20	*A. gossypii*	Few C	–
S50	Pot	Eggplant	DW	250	100	2017/10/20	*A. gossypii*	Few C	–
S30	Pot	Eggplant	TW	250	100	2017/8/11	leafminer*^5^	–	–
S28	Pot	Eggplant	TW	250	100	2017/8/11	leafminer*^5^	–	–
S29	Pot	Eggplant	TW	250	100	2017/8/11	leafminer*^5^	–	–
S31	Pot	Eggplant	TW	250	100	2017/8/11	leafminer*^5^	–	–
S32	Pot	Eggplant	TW	250	100	2017/9/5	leafminer*^5^	–	–
S33	Pot	Eggplant	TW	250	100	2017/9/5	leafminer*^5^	–	–
S34	Pot	Eggplant	TW	250	100	2017/9/5	leafminer*^5^	–	–
S35	Pot	Eggplant	TW	250	100	2017/9/5	leafminer*^5^	–	–
S22	Pot	Cabbage	TW	250	100	2017/7/14	*B. brassicae*	102	–
S23	Pot	Cabbage	TW	250	100	2017/7/14	*B. brassicae*	63	–
S05	Pot	Cabbage	TW	250	100	2017/6/30	*B. brassicae*	1 C	–
S13	Pot	Cabbage	TW	250	100	2017/7/7	*B. brassicae*	1 C	–
S14	Pot	Cabbage	TW	250	100	2017/7/7	*B. brassicae*	2 C	–
S06	Pot	Cabbage	TW	250	100	2017/6/30	*B. brassicae*	2 C	
							*Sphaerophoria* sp	1	Larva
S08	Pot	Cabbage	TW	250	100	2017/6/30	*M. persicae*	1 C	–
S16	Pot	Cabbage	TW	250	100	2017/7/7	*M. persicae*	1 C	–
S24	Pot	Cabbage	TW	250	100	2017/7/14	*M. persicae*	28	–
S07	Pot	Cabbage	TW	250	100	2017/6/30	*P. rapae*	1	Egg
S15	Pot	Cabbage	TW	250	100	2017/7/7	*P. rapae*	1	Egg
S26	Pot	Cabbage	TW	250	100	2017/7/14	*P. rapae*	1	Larva
							*P. xylostella*	1	Larva
S25	Pot	Cabbage	TW	250	100	2017/7/14	*P. rapae*	1	Pupa
S09	Pot	Cabbage	TW	250	100	2017/6/30	*P. rapae*	1 2	Egg JH larvae*^8^
S17	Pot	Cabbage	TW	250	100	2017/7/7	*P. xylostella*	1	Larva
S04	Pot	Cabbage	TW	250	100	2017/6/30	Unobserved chewer	–	
S12	Pot	Cabbage	TW	250	100	2017/7/7	Unobserved chewer	–	
S21	Pot	Cabbage	TW	250	100	2017/7/14	Unobserved chewer	–	
S10	Field	Cabbage	TW	4000	100	2017/7/4	*B. brassicae*	12	
							*M. persicae*	3	
							*P. rapae*	4, 19, 2	Egg, larva, pupae
							*P. xylostella* or P. rapae*^6^	6	Larvae
S11	Field	Cabbage	TW	4000	100	2017/7/4	*P. rapae*	1 5	Egg larvae
							*P. xylostella*	7	Larvae
S18	Field	Cabbage	Rainfall	–	30	2017/7/7	*P. rapae*	2	Larvae
							*P. xylostella*	10	Larvae
S19	Field	Cabbage	Rainfall	–	40	2017/7/7	*P. rapae*	2	Larvae
							*P. xylostella* or P. rapae*^6^	2	Larvae
S20	Field	Cabbage	Rainfall	–	45	2017/7/7	*P. rapae*	2	Larvae
							*P. xylostella*	3	Larvae

*^1^Tree eggplants were used for sampling.

*^2^Either tap water (TW) or distilled water (DW) was used to collect eDNA.

*^3^Target species were observed or artificially introduced into a plant.

*^4^The *A. gossypii* was mummy (parasitized by aphid parasitoid).

*^5^We only checked if there were leaf miner tracks, but not if the insect itself was in it.

*^6^We could not distinguish between *P. xylostella* and *P. rape* because they were too small to observe.

*^7^The number of herbivores on the plant was counted on the sampling day. In some cases, we did not count the number of aphids but counted the colony (C) of aphids. “Few C” means the presence of a few colonies, but we did not count the exact number of colonies. “– “Shows that we did not introduce or observed any herbivore.

*^8^JH larvae indicate larvae hatched from eggs.

We detected several non-target species (i.e., individuals of several species could not be visually identified) in the samples collected from eggplants by both tap or distilled water (Table S3). eDNA metabarcoding also detected major pest arthropods such as a thrip, *Thrips tabaci* (8/12 tap water samples and 1/2 distilled water samples, Table S3), a spider mite, *Tetranychus* (10/12 tap water samples and 1/2 distilled waters samples, Table S3) and tarsonemid mites, Tarsonemidae (4/12 tap water samples and 2/2 distilled water samples, Table S3). These herbivores are rarely controlled by tiny pests in agriculture because of their low visibility.

### eDNA collected from potted cabbage

The eDNA of the target species *B. brassicae* and *M. persicae* was detected in water samples from potted cabbage plants (4/6 and 3/3 samples, respectively; Table 5). In many samples from which *B. brassicae* or *M. persicae* were detected, aphid parasitoids were also detected *Diaeretiella rapae* (4/4 samples detected *B. brassicae*) and *Aphidius colemani* (2/4 samples detected *B. brassicae* and 1/3 samples detected *M. persicae*) (Table 5). eDNA of *Sphaerophoria macrogaster* was detected in one sample collected from the cabbages, on which *S. macrogaster* was visually observed together with *B. brassicae*.

**Table 5:** The number of target species, *Brevicoryne brassicae, Myzus persicae*, and *Sphaerophoria macrogaster* observed on a potted cabbage at the sampling day and total sequence reads of these target species and the parasitoids, *Diaeretiella rapae* and *Aphidius colemani*, of these aphid species detected from samples collected by “plant flow collection”.

	Target species			Parasitoid of the target species	
Sample ID	Species	The number	Sequence reads	Sequence reads of *D. rapae*	Sequence reads of *A. colemani*
S5	*B. brassicae*	C*	0	0	0
S13	*B. brassicae*	C*	19,907	4441	8
S14	*B. brassicae*	C*	2	1	3
S22	*B. brassicae*	102	254	9326	0
S23	*B. brassicae*	63	362	896	0
S6	*B. brassicae*	C*	0	0	0
	*S. macrogaster*	1	18	–	–
S8	*M. persicae*	C*	40,886	0	0
S16	*M. persicae*	C*	1	0	0
S24	*M. persicae*	28	17	0	6

Tap water was used to collect eDNA. The amount of water and sampling day was described in Table 3.

*The number of aphids was not counted, but we recorded that there were a few colonies (C).

*P. rapae* was detected in eDNA samples collected from potted cabbages on which either an egg together with two freshly hatched larvae, three larvae, a larva or a pupa respectively was observed (S9, S17, S26, and S25, respectively, Table 6). However, in samples containing a larva, an egg, or an egg together with two freshly hatched larvae, respectively (S15 and S7, respectively, Table 6) no proof of occurrence was found in eDNA samples. *P. xylostella* was detected in eDNA samples (1/2 samples, Table 6). The detection probabilities of *P. rapae* eDNA would depend on the developmental stage, body size, and number of individuals on the plant as the recent study shows the shifts of arthropod diversity^17^.

**Table 6:** The developmental stage of target herbivorous species, *Pieris rapae* and *Plutera xylostella* observed on a potted cabbage at the sampling day and their sequence reads detected from 100 ml water samples collected from 250 ml tap water sprayed on plant surface by “plant flow collection”.

Sample ID	Target species	The number of individuals	Developmental stage	Sequence reads
S7	*P. rapae*	1	Egg	0
S9	*P. rapae*	1	Egg	1
		2	& JH larvae*	
S15	*P. rapae*	1	Larva	0
S17	*P. rapae*	3	Larva	1269
	*P. xylostella*	1	Larva	0
S26	*P. rapae*	1	Larva	3
	*P. xylostella*	1	Larva	6
S25	*P. rapae*	1	Pupa	20

*JH indicates larvae hatched from eggs.

Visually unobserved arthropods were detected in eDNA samples, which were collected from potted cabbage plants. *T. tabaci* was found the most frequently (9/12 samples, Table S3). eDNA from the other species, chrinomid *Cladotanytarsus vanderwulpi*, fruit fly *S. pallida*, leaf miner *L. sativae*, grasshopper *Tetriginae* japonica. booklice *Cerobasis guestfalica*, mealybug *Planococcus citri*, were detected once in 12 potted cabbages except for mealybug *Planococcus citri*, which was found in two samples (Table S3), in addition to the target species. Target species, *P. xylostella*, *P. rapae*, *M. persicae*, and *B. brassicae*, were detected in some samples on which they were not visually observed. Similarly, several arthropods, such as *T. tabaci, B. brassicae,* and *Liposcelis,* were detected in eDNA samples collected from the surface of cabbage with chewing damage, on which no arthropods were visually observed (Table S4). eDNA of chewing herbivores were successfully detected in three samples collected from potted cabbages, on which chewing damages existed but the chewing herbivores were not directly observed (S04 and S12: *P*. *rapae*, S21: *Tetrix japonica*, Table S4). This result confirmed that eDNA of arthropods can be detected even in the absence of the herbivore on the plant when collecting the eDNA as recent studies has shown^16,20^. However, reads of detected species in S04 and S21 were singletons (Table S4). This suggests that most of eDNA, which had existed on surfaces of the cabbage plants would be washed by watering and/or degraded by the other environmental factors^26,27^.

### eDNA collected from field cabbage by using tap water and rainfall

**Figure 1:**
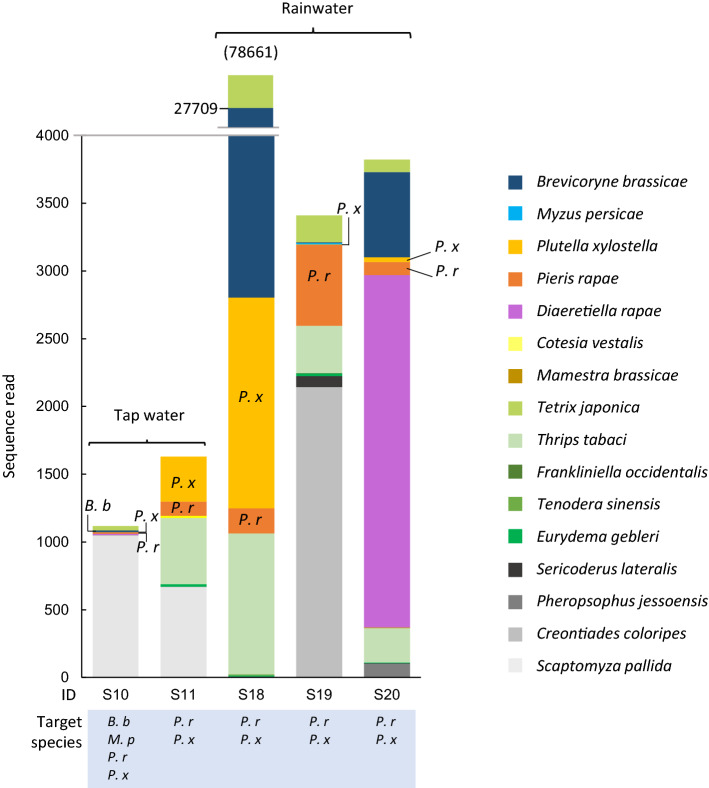
The sequence reads of species detected from field cabbage. They were detected from eDNA samples collected from the surface of field cabbage by tap water (S10 and S11) or rainfall (S18-20) and including data identified at species level. The other sequences identified at family or genus level are also described in Table S3. Label of under each bar is sample ID and the target species of each sample, *B. b*: *Brevicoryne brassicae*, *M. p*: *Myzus persicael*, *P. r*; *Pieris rapae*, *P.x*: *Plutella xylostella*. When target species were detected in a sample, the abbreviation of species name is described in a graph. The value in the parenthesis is the total sequence reads of S18. The number of target species observed on a sampling plant is described in Table 4.

Total 10 and 16 arthropod species were detected in the eDNA samples collected from the surface of cabbage grown in the field by either tap water or rainfall (Fig. 1). Among these species, visually observed target species, such as *B. brassicae*, *P. rapae*, and *P. xylostella* were all successfully detected in both tap water and rainfall samples (*B. brassicae*: 1/1 tap water sample, *P. rapae*: 2/2 tap water and 3/3 rainfall samples, and *P. xylostella*: 2/2 tap water and 3/3 rainfall samples, Fig. 1). Although the total read number showed large variations between these samples (from 3,035 to 281,846), the sampling coverage was greater than 98%). So, the standardized result (Fig. S2) was qualitatively similar to the unstandardized result (Fig. 1) (see Coverage-based methods and results in SI).

Because it was very difficult to distinguish young larvae of *P. rapa* and *P. xylostella* by eye*,* this method would contribute to identify the species of lepidoptera larvae on a plant. In addition, we could not visually assess parasitoid adults due to the difficulty in species identification and their short staying time on a plant. However, we detected eDNA of many parasitoid species in both tap water and rainfall samples collected from cabbage in a field. eDNA of aphid parasitoids *D. rapae* were detected together with eDNA of its host *B. brassicae* in two samples (1/2 tap water and 1/3 rainfall samples, Fig. 1). This result was similar to that of potted cabbages (Table 5). A larval parasitoid of *P. xylostella, Cotesia vestalis* was detected in one tap water eDNA sample together with eDNA of *P. xylostella* among five samples with *P. xylostella* (Fig. 1). Other parasitoids, an egg parasitoid, *Trichogramma*, and a parasitoid of the leaf miner fly, *Kleidotoma* were detected in one tap water sample and in one rainfall sample, respectively (Table S3). These eDNA from parasitoids remained probably in their feces and urine on plants. In addition, eDNA would be present in the chemical cues on the surface of host bodies secreted by parasitoids when laying eggs. The parasitoids’ eDNA would remain inside the host body that they had killed and emerged from, and the parasitoid’s cocoon residuals. These remaining eDNA can get into the water samples.

Other visually unobserved species, which were detected in both eDNA samples collected by tap water and rainfall, were an onion thrip *T. tabaci* (in all samples including tap and rain water samples)*,* grasshopper *Tetrix japonica*, shield bug *Eurydema gebleri* (1/2 tap and 3/3 rain water samples), fruit fly *Scaptomyza pallida* (2/2 tap, 1/3 rain water samples), and plant bug *Creontiades coloripes* (1/2 tap, 1/3 rain). Total read of *T. japonica* in all five samples collected in field was the most abundant in the identified arthropods at least family level. Another four species, a western flower thrip *Frankliniella occdentalis* lepidopterans *Manestra brassicae* and *Bradina diagonalis,* and a predator, mantis *Tenodera sinensis* (Fig. 1), were only detected in a rainfall sample. Predators, spider, *Leucauge* sp. and predatory mite *Euseius* sp., which were identified at genus level, were detected in a rainfall sample (1/3 rainfall samples) in addition to *T. sinensis* (Table S3)*.*

We did not find any evidence that *Eurydema gebleri* and *Bradina diagonalis* exist in Japan. The DNA-based identification of *E. gebleri* might be mistaken because there is a subspecies, *E. rugose*, which is present in Japan. These two subspecies are closely related each other and the alignment of their sequences resulted in 99% identity with 0.0 E-value. Such a high similarity is one of the reasons for the wrong assignment of species identity. To be worse, the sequences of *E. rugose* in NCBI were short and did not cover the whole sequenced region in this study, resulting in the greater E-values than those of *E. gebleri* when aligned to our reads. Therefore, even with the greater identity of our reads against *E. rugose* (100%) than against *E. gebleri* (99%), *E. gebleri* was the top hit in BLAST search for our reads. Furthermore, only two sequences of *E. rugose* are available in NCBI, of which one was deposited from Japan and the other was from Korea, while 55 sequences of *E.gebleri* were available in NCBI. Since the QCauto method^28^ relies on the top hit of NCBI blast search against a query sequence (i.e., our reads) and the taxonomic consistency of the local neighborhood sequences in the NCBI references, the misidentification of our reads as *E.gebleri* was due to the lower E-value and greater availability of *E. gebleri*’s sequences than those of *E.rugose.* Similarly, because the sequences of *Bradina* species present in Japan were rarely available in NCBI, the result of assignment to the sequence that was identified as *B. diagonalis* might not be reliable. These misidentifications illustrate an urgent need for improving the size and completeness of the global and local DNA barcode libraries to avoid false positive results as well as false negative ones^25^.

### Detection performance of arthropod eDNA

The detection performance of arthropod eDNA in our method was comparable to recent studies with similar methods in terms of the species richness detected. In this study, we successfully detected eDNA of 42 additional species of which taxonomy was assigned at least to the genus level in overall 37 samples in addition to seven target species (Table S3) from two plant species only without harvesting any plant tissues. One study collecting rainfall under the canopies of four tree species with two replications for each species detected eDNA of 50 invertebrate species^19^. Another study collecting 56 individual flowers from seven plant species detected eDNA of 135 arthropod species in 67 families^16^. Unlike these studies, we were also able to calculate eDNA detection rates because we artificially introduced or directly observed herbivores on plants before collecting water and sampling eDNA. The detection rate was quite high 82% when we excluded the cases when eDNA were detected from plants without feeding herbivores but with feeding track (Table 2).

There are two possible reasons for the detection of several arthropod species that were not visually observed on plants when the eDNA samples were collected. First, arthropods are simply too small and overlooked. The second reason is that they had existed on the plant previously and only eDNA remained on the plant (see Valentin et al*.*^25^ for the persistence of eDNA on plants). In future studies, it will be necessary to investigate the “ecology” of eDNA (e.g., how it is released, moved, and persists on a plant surface), which is currently being extensively studied in fish eDNA studies^29,30^. In a recent study, eDNA of ungulate species was detected in 50% of ungulate species browsed twigs even after 12 weeks^21^. However, arthropods would release much less amount of their DNA on plants, so the eDNA of arthropods would become undetectable much earlier than that of ungulate species even with the same DNA degradation rate.

### The possibility that rainfall collecting eDNA of arthropods included aerial eDNA

A recent study demonstrated that arthropod eDNA can be collected from air samples in a field condition^18^. Such aerial eDNA could deposit on plant surface so that eDNA collected from surface of cabbage by rainfall could include it. Our cabbage field was very small (6 × 5 m) and the other crops such as rice, blueberry, strawberry, tomato, and plum were growing at a few meters away from there. However, except for *B. diagonalis,* our results rarely included unexpected arthropod species, which would not be associated with cabbage plants but included in rain fall samples. The amount of air collected to detect aerial eDNA from arthropods in the previous study is much higher (6000–9000 L in 20–30 min^18^) than that of rainfall collected in present study (30 to 45 mL). However, we should carefully treat the result from rainfall because not only aerial eDNA of arthropods but also microbial DNA are included in precipitation, which can be transported across seas^31^. Thus, a further study is necessary to investigate how often and how much aerial arthropod eDNA and microbial DNA are mixed with local arthropod eDNA originated from the plant surface. Then, we will be able to evaluate the false positive probability that eDNA of pest species detected by our method comes from a field nearby where crops are infested with it but not in the field under investigation.

### Pros and cons for using three types of water for plant flow method

When using tap water, we should be very careful. Tap water could contain bacteria and eDNA remains from eukaryotes. Tap water must be conformed to satisfy the water quality standards, which are specified in the "Ministerial Ordinance on Water Quality Standards" in accordance with the provisions of the Water Supply Law in Japan. In the standards, the number of bacterial colonies recovered from 1 ml test water should be less than or equal to 100. The annual report of the local government (Nara City Enterprise Bureau) tells that the monthly survey for three years at 12 sites of bacterial contamination using the standard agar medium incubation at 12 resulted in seven colonies at maximum but even a single bacterial colony was not detected from most of the tested samples. As well as such high quality of tap water used in our experiment, the pre-sterilization of all equipment by 1.0% sodium hypochlorite solution probably excluded most parts of any minor eDNA contamination.

In addition to the possibility of contamination of eDNA, tap water usually contain residual chlorine (in Japan, less than 1 mg/L) and would degrade eDNA collected from plant surface by plant flow method. However, the distilled water is more expensive than tap water and not easily available for citizen scientists. Furthermore, farmers would benefit greatly if it were possible to conduct eDNA-based pest monitoring while also watering crops. In Japan, river water and groundwater are commonly used for agricultural purposes. However, these water sources may inhibit to detect and identify arthropod eDNA from plant surfaces due to the presence of large amounts of arthropod eDNA in river water^32^ and bacterial eDNA in groundwater^33^. Furthermore, the use of distilled water for agricultural purposes may also have negative effects on plant growth. Distilled water has strong solubility, which can result in the depletion of essential minerals from plants over time and may potentially affect plant growth. Therefore, it may be preferable to use tap water for collecting eDNA in fields and other agricultural areas once it is confirmed that the tap water quality is enough for the plant flow method in the focal regions before designing monitoring program.

To use rainfall for our method has contamination risk from air and precipitation itself, which we discussed above section. In addition to that, periodic survey using our method by rainfall is difficult at a region of low precipitation and during low precipitation periods. However, plant flow collection method using the rainfall would make a great contribution to the study of arthropods associated with tall trees, to which it is difficult to spray water to a whole plant body artificially, as Macher et al.^19^ demonstrated. In areas such as tropical rainforests, where rain falls regularly and tall trees are dense, using rainfall would be recommended for our method. However, we recommend confirming how often contamination occurs from air by collecting rainfall itself as well as tree stem flow when rainfall is used to collect eDNA.

We also evaluated the performance of three types of water in terms of the estimated ASV richness. However, it is important to interpret the following statistical results with caution due to the limited sample size and uneven distribution of sequence size among the replicates and water types. At the same time, it is also notable that sample coverage was relatively high (> 90%), indicating that our sampling effort was sufficient to capture most of the ASV richness independently of water types and the sequence sizes. In addition, the sample-coverage-standardized estimator^34^ provides a more reliable comparison of the ASV richness among the water types, although the use of such an estimator does not completely eliminate the effect of the variation in the number of sequences between samples. The averages (95% confidence interval) of the estimated ASV richness with the standardized sample coverage (98.5%) are 108.9 ([-29.29117, 247.0779]) and 325.9 ([289.8204, 362.0253]) from the tap water (n = 2; sequence size was 3,035 and 103,346 reads, respectively) and rainfall (n = 3; sequence size ranged from 146,901 to 281,846 reads) samples, respectively, for the field cabbage data. Then, the average of the estimated ASV richness was statistically greater from the rainfall samples than tap water samples (linear model, P = 0.000527). Similarly, the averages (95% confidence interval) of the estimated ASV richness with the standardized sample coverage (92.3%) was 10.5 ([3.495311, 17.40478]) and 7.8 ([-13.44648, 29.03981]) from the tap water (n = 12; sequence size ranged from 103 to 31,396 reads) and distilled water (n = 2; sequence size was 170 and 1,007, respectively) samples, respectively, for the eggplant data. Then, the average of the estimated ASV richness with was not statistically different (linear model, P = 0.747) between tap and distilled water.

We would like to note that making a direct comparison of sequence sizes and ASV richness between various samples, especially those from different growing conditions (field, glass house, and climate chamber), is misleading. This is because other factors can affect the results, such as the size and developmental stage of the plants, and the accessibility of arthropods. For example, the cabbages in the field had heads and were larger than the potted cabbages with only five leaves in the glass house. In the field, arthropods could easily visit the cabbages, while in the glass house, some arthropod immigration was possible. However, in the climate chamber, only inoculated insect species remained on the plants (in case of eggplants). Therefore, the differences in the sequence size and ASV richness were not necessarily due to the differences in the water types.

### Possible reasons for different richness between tap water and rainfall samples

There are several possible explanations for why the estimated richness at the ASV level in rainfall samples was significantly greater than in tap water samples. The first one is the above mentioned influence of aerial arthropod eDNA on the rainfall samples. The second is the longer collection time; rainfall was collected over night while spraying tap water on a cabbage took approximately five minutes only. This could significantly increase the possibility that eDNA attached to the plant get washed in. The third is that some arthropods drinking water from the dust pans would contribute to an increase in the eDNA collected in rainfall. Because the color of dust pans used in the field experiment was gray and several arthropods inhabiting the ground strata which are not attracted by color, the effect of color to attract arthropods would not be high.

Finally, we would mention the other potential mechanism. The recovery rate of water (ratio of the filtered water to the sprayed tap water) in tap water samples was substantially lower than the rate (ratio of filtered water to the precipitation) in rainfall samples. The amount of water filtered by a single Sterivex filter cartridge was much less (100 ml) than the amount of tap water sprayed (4,000 ml). The other fractions of the sprayed tap water ran off around the dustpans used for collecting water and also accumulated inside the plant due to the headed shape of cabbages. It was likely that the recovery rate of eDNA was also low given the low recovery rate of water (less than 10%). On the other hand, despite the fact that the amount of filtered water (approximately 40 mL) was less than that of tap water (100 ml), the actual amount of rainfall was not that high, and it is likely that the rain fell slowly on the plant’s surface before reaching the base of the shoots. It follows that the loss of eDNA during the collection steps was expected to be low, leading to a high recovery rate of eDNA and a greater estimate of ASV richness. Therefore, it will be better to reduce the amount of water sprayed as much as possible, wash the entire plant body slowly, and take more time when using tap water or distilled water for washing the plant surface, in order to improve the water recovery rate and subsequently the eDNA recovery rate.

## Conclusion

**Figure 2:**
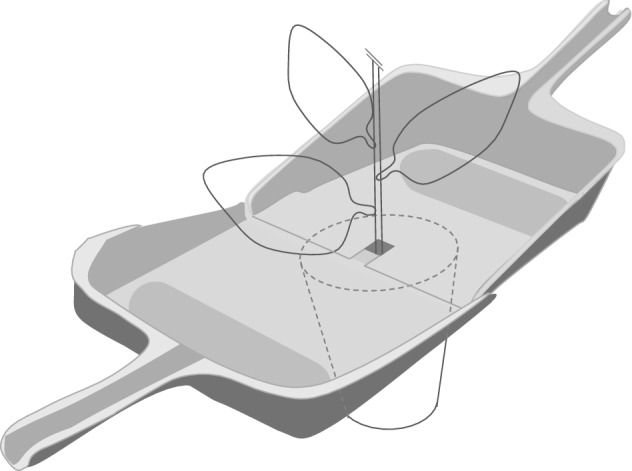
Pattern diagram of the “plant flow collection” method. A set of two dustpans connected to each other was used. Dustpans were placed across the base of the plant stem on the soil.

In this study, we developed a non-destructive method for collecting eDNA from whole plants by water spraying or using rainfall. The method was able to detected a wide range of arthropods, including not only conspicuous herbivores, but also parasitoids, predators, and meiofaunal organisms, which are difficult to detect visually, from whole plant bodies with a single sample collection. We can conclude that our method is possible to detect eDNA from plants that harbor diverse arthropod species. It is also worth noting that the combination of eDNA sampling with direct observation or artificial introduction of arthropods on plants enabled us to evaluate the detection rate of eDNA. In some cases, small insects such as aphids or spider mites themselves may be included in the collected water because they were too small for us to easily notice their presence in water. Thus, we could not exclude the possibility that drowning small arthropod, which would release eDNA in water, influencing the number of reads in samples. However, including arthropod individuals themselves in sampling water is not a matter for monitoring when our purpose is to know the presence and absence of organisms on a plant. It is necessary to further investigate the differences in detection probabilities among different taxa of arthropods, number of individuals, and feeding behaviors. At the same time, our study clearly shows that, by applying our method of “plant flow collection” (i.e., collection of sprayed water or rainfall at the bottom of a plant, Fig. 2), it is possible to detect small arthropods that are often overlooked, natural enemies of pest arthropods, such as parasitoids (i.e., *D. rapae* and *C. vestalis*) that are not easily observed visually, leaf miners that are hidden in leaves, and grasshoppers that are highly mobile. Therefore, our non-destructive method would be widely applicable for surveying pests and natural enemies in pest management and monitoring arthropods in plant species.

## Methods

### Treatment for plants

Our study complied with the relevant institutional, national, and international guidelines and legislation. The present research did not include any collection of plant material and involve any species at risk of extinction and the convention on the trade in endangered species of wild fauna and flora.

Seeds of eggplants *Solanum melongena* L. (cv. Senryo nigo) and cabbage *Brassica oleracea* L. var. capitata L. (cv. YR50) were purchased from a commercial supplier (Takii & Co., Ltd., Kyoto). Those plants were cultivated individually in pots (Φ 7 cm, 9 cm high) filled with culture soil from seeds in a glass house (25 °C ± 10 °C) located in a common garden of Faculty of Agricultre, Kindai University, Nara, Japan (34.6694 N, 135.7381E). We used potted eggplant and cabbage with approximately five leaves for the laboratory experiments. Cotton aphids, *Aphididae gossypii* Glover, from a colony maintained in our laboratory (Faculty of Agriculture, Kindai University, Nara, Japan, 34.6694 N, 135.7381E) were placed on the leaves of an eggplant (5, 10, or 50 aphid individuals on each plant) in a transparent plastic box (25 cm × 30 cm × 28 cm) in a climate chamber (25 ± 3 °C, 16:8 h light: dark cycle) and kept for 7 d (the final number of aphids on a plant was 39, 50, and 157, respectively). We also used nine potted eggplants that were selected in a glass house. One of the eggplants had five cotton aphids, which became mummy, and the other eight had no herbivore but only the feeding tracks of leaf miners.

We selected potted cabbage in a glass house infested naturally by white butterfly *Pieris rapae* L*,* diamond back moth *Plutella xylostella* L.*,* green peach aphid *Myzus persicae* Sulzer, or cabbage aphid *Brevicoryne brassicae* L., kept in a plastic case (27 × 15 × 13 cm), and collected eDNA from plant surfaces with these insects. We also used potted cabbage with only the feeding tracks of chewers (N = 3) to test whether herbivores could be detected only from the feeding tracks. The sampling days are listed in Table 4.

Forty-eight cabbage plants with approximately five leaves per plant were transplanted in a field along with six lines. There was a 1 m interval between lines, and there were eight plants in each line with a 50 cm interval between plants in a common garden of the Faculty of Agriculture, Kindai University in Nara, Japan, in May 2017. Five randomly selected cabbages in the cabbage field were used for the experiments when they formed heads on July 4th and 7th, 2017.

Seven arthropod species, *A. gossypii, P. rapae, P. xylostella, M. persicae, B. brassicae,* a leaf miner fly (Agromyzidae), and *S. macrogaster*, which were artificially introduced or visually observed on an experimental plant, were hereafter termed “target species” in this study.

### Collection of eDNA

All sampling equipment was sterilized with a 1.0% sodium hypochlorite solution before use. A dustpan (1–4 cm depth × 25 cm wide × 20 cm with a 12 cm handle) with a cut (2 cm × 5 cm) opposite to the handle, where the stem of a cabbage or eggplant was in, and another uncut dustpan were connected to it (Fig. 2) and covered with a polyvinylidene chloride lap to close the gap between the hole of the dustpan and the stem. Similarly, two dustpans covered with polyvinylidene chloride lap were inserted under cabbage planted in the field. In order to disseminate this technique to detect pest insect attacks and apply it to large-scale biodistribution surveys (e.g., with citizen participants), we tested tap water for a simplified process in addition to distilled water for collecting eDNA. Tap water had been distilled with polyaluminium chloride, sodium hydroxide, sodium hypochlorite at water purification plant in Japan. pH and residual chlorine were kept about 7.5 and less than 1 mg /L respectively at hydrant in Japan. Tap or distilled water was sprinkled throughout the plant body. Two-hundred fifty ml tap water or distilled water was used for each potted plant by a 250 mL wash bottle and 4 L tap water was sprayed using a 2 L watering can with a rose sprinkler head or rainfall were used for field cabbage (Table 4). Five-hundred mL of distilled water was sprayed and 350 mL distilled water flow through the surface of three eggplants infested by aphids was collected as a eDNA sample to increase the collection rate (Table 4). The sprayed water ran through the whole plant surface from the leaves to the stems, and water was gathered on the dustpan. Rainwater pooled over a day was collected one day after the dustpan was set under the cabbage. Details of the sample information, such as sampling day, amount of poured and filtered water, and the number and developmental stage of herbivores observed on a plant, are described in Table 4. We refer to this method as “plant flow collection”.

We collected 30–350 ml water (Table 4) pooled in dustpans by sacking with a plastic disposable syringe (SS-50LZ, Terumo Co.) and filtered it with φ0.45 µm Sterivex™ filter cartridges (SVGV010RS, Merck Millipore, Darmstadt, Germany). The amount of sprayed water (tap water or distilled water, Table 4) was adjusted depending on plant sizes, resulting in the variations in the amount of the collected water (80–350 ml, Table 4). When the rainfall was used for collecting water, the amount of the collected water (30–45 ml, Table 4) simply depended on the amount of precipitation. Two milliliters of RNAlater solution were added to the filter cartridges and stored at − 60 °C until further processing.

We also collected aerosol instead of washing processes as field-negative controls. The suction port of the air pumps (Air Sampler Mini Pump MP-Σ100HNII, SIBATA SCIENTIFIC TECHNOLOGY LTD) was connected to the outlet of φ0.45 µm Sterivex™ filter cartridge using polytetrafluoroethylene tube. The Sterivex cartridge was placed with its inlet facing downwards at a height of 2 m above the floor in the center of the glass house where the plants used for the experiment were grown. Three replicates were collected for a sampling duration of 3 h each, while two replicates were collected for a sampling duration of 48 h each, at a flow rate of 100 ml/min, resulting in a total sampling volume of 18 l and 288 l of air per sample, respectively. These field-negative controls are reasonably used for evaluating and normalizing contaminations at the extraction step as well as those from aerial eDNA.

### DNA extraction

The RNAlater solution in each filter cartridge was removed by vacuum (EZ-Vac Vacuum Manifold) and then washed with 1 ml of MilliQ water without mechanically destroying the filter cartridge for reducing the risk of contamination; a recent study demonstrates that this method does not significantly reduce the amount of eDNA extracted compared with the method that destroys the filter cartridge^35^. DNA was extracted from cartridge filters using a DNeasy® Blood & Tissue Kit (QIAGEN, Hilden, Germany) referring to the method described by Miya et al*.*^36^. Briefly, proteinase K solution (20 µL), PBS (220 µL), and buffer AL (200 µL) were mixed and 440 µL of the mixture was added to each filter cartridge. The materials on the cartridge filters were lysed by incubating on a rotary shaker (15 rpm) at 56 °C for 10 min. The mixture was transferred into a new 2 ml tube from the inlet of the filter cartridge by centrifugation (3,500 × g for 1 min). The collected DNA was purified using a DNeasy® Blood & Tissue Kit following the manufacturer’s protocol. After purification, the DNA was eluted using 100 µL of elution buffer provided with the kit. The eluted DNAs was stored at – 20 °C until further processing.

### First and second PCR

Prior to library preparation, the workspaces and equipment were sterilized. Filtered pipette tips were used, and separation of pre- and post-PCR samples was carried out to safeguard against cross-contamination. Two negative controls (PCR-negative controls) were used to monitor contamination during the experiments. For the first PCR, 6 µL KAPA HiFi HotStart ReadyMix (KAPA Biosystems, Wilmington, WA, USA), 0.7 µL primer (5 µM of each primer; Forward, mlCOIIntF; Reverse, HCO2198^37^) with adaptor and six random bases (Table S5), 2.6 µL sterilized distilled H_2_O, and 2 µL DNA template. The thermal cycle profile after an initial 2 min denaturation at 95 °C was as follows (35 cycles): denaturation at 98 °C for 20 s, annealing at 52 °C for 30 s, and extension at 72 °C for 30 s, with a final extension at 72◦C for 1 min. The first PCR product was purified using AMPure XP (PCR product: AMPure XP beads = 1:0.8; Beckman Coulter, Brea, California, USA). We performed a duplicate 1st PCR. These products were pooled to reduce PCR dropouts, diluted tenfold, and used as templates for the second PCR. 2nd PCR was performed with 24 µl reaction volume containing 12 µl of 2 × KAPA HiFi HotStart ReadyMix, 1.4 µl of each primer (5 µM of each primer, Table S6), 7.2 µl of sterilized distilled H_2_O and 2.0 µl of template. Different combinations of forward and reverse indices were used for different templates (samples) for massive parallel sequencing with MiSeq (Table S7). The thermal cycle profile after an initial 2 min denaturation at 95 °C was as follows (12 cycles): denaturation at 98 °C for 20 s, annealing at 60 °C for 30 s, and extension at 72 °C for 1 min, with a final extension at 72 °C for 1 min.

Each 20 µL second PCR product was pooled. The pooled library was purified using AMPure XP (PCR product: AMPure XP beads, 1:0.8). Appropriately 500 bp libraries were size-selected using 2% E-Gel Size Select (Thermo Fisher Scientific, Waltham, MA, USA). The double-stranded DNA concentration of the library was quantified using a Qubit dsDNA HS assay kit and Qubit fluorometer (Thermo Fisher Scientific, Waltham, MA, USA). The double-stranded DNA concentration of the library was adjusted to 4 nM using Milli-Q water, and the DNA was applied to the MiSeq platform (Illumina, San Diego, CA, USA). Sequencing was performed using a MiSeq Reagent Nano Kit v2 for 2 × 250 bp PE (Illumina, San Diego, CA, USA). The sequence depth of each sample was described in Table S7.

### Sequence data processing

All pipelines used for sequence read processing and taxonomic assignment followed Ushio^35^. Briefly, the raw MiSeq data were converted into FASTQ files using the bcl2fastq program provided by Illumina (bcl2fastq v2.18), and the FASTQ files were demultiplexed using Claident v0.2.2018.05.29 (http://www.claident.org^28^). Demultiplexed FASTQ files were analyzed using the Amplicon Sequence Variant (ASV) method implemented in DADA2 v1.7.0^38^. To filter the quality, forward and reverse sequences were trimmed to lengths of 240 and 200, respectively, based on the visual inspection of the Q-score distribution using the DADA2::filterAndTrim() function. The error rates were learned using the DADA2::learnErrors() function. Sequences were then dereplicated, error-corrected, and merged to produce an ASV-sample matrix. All samples were pooled together for error corrections with dada(…, pool = TRUE) in order to avoid the deletion of singletons and doubletons in each sample. Taxonomic identification was performed for ASVs inferred using DADA2, based on the query-centric auto-k-nearest-neighbor (QCauto) method^28^. Using Claident v0.9.2021.10.22, taxonomic assignment was conducted with the lowest common ancestor algorithm^39^; a taxonomic unit is assigned to the query (i.e., our reads) to the lowest taxonomic level where all of the nearest-neighbor and the neighborhood sequences are consistent^28^. The “overall_genus” database (database containing all sequences in NCBI nt with genus or lower-level taxonomic information), which was prepared in Claident, was used for taxonomic assignment. Arthropods were the focus of this study (Table S3). When the performance of the proposed method was quantitatively compared, the sample coverage (as the index of sampling effort^34^) was first estimated and then the coverage-based richness (the number of distinct types) of ASVs was estimated with the common sample coverage among the focal samples using the iNEXT::DataInfo() function. When the species composition was compared with the standardized coverage, the ASV-sample matrix was resampled and rarefied using resample() function and then the same procedure was conducted for taxonomic assignment.

## Supplementary Information

Supplementary Information 1.

Supplementary Table S2.

Supplementary Table S7.

Supplementary Table S8.

Supplementary Table S9.

Supplementary Table S10.

## Data Availability

DDBJ Accession numbers of the DNA sequences analyzed in the present study are DRA014843 and DRA015707 (Submission ID) and DRR404932-DRR404970 and DRR443406-DRR443410 (Run ID).
