# Supplementary material for: Non-destructive collection and metabarcoding of arthropod environmental DNA remained on a terrestrial plant
Source: Sci Rep. 2023 May 12;13:7125. doi: 10.1038/s41598-023-32862-4 (PMC10182007; doi:10.1038/s41598-023-32862-4)
Supplement: Supplementary file 1 — Supplementary Information 1. [file 41598_2023_32862_MOESM1_ESM.docx]

**Supplementary information**

**Title**

Non-destructive collection and metabarcoding of arthropod environmental DNA remained on a terrestrial plant

**Authors**

Kinuyo Yoneya^1,2*^, Masayuki Ushio^3,4,5*^, Takeshi Miki^2,6^

^1^Faculty of Agriculture, Kindai University, 3327-204, Nakamachi, Nara 631-8505, Japan.

^2^Center for Biodiversity Science, Ryukoku University, 1-5 Yokotani, Seta Oe-cho, Otsu, Shiga 520-2194, Japan.

^3^Hakubi Center, Kyoto University, Kyoto, 606-8501, Japan.

^4^Center for Ecological Research, Kyoto University, Otsu, 520-2113, Japan.

^5^Department of Ocean Science, The Hong Kong University of Science and Technology, Clear Water Bay, Kowloon,

Hong Kong SAR, China

^6^Faculty of Advanced Science and Technology, Ryukoku University, 1-5 Yokotani, Seta Oe-cho,

Otsu, Shiga 520-2194, Japan

Contents:

(Supplementary methods & results) **Coverage-based methods and results**

Figure S1. The total sequence reads of leaf-miner flies, Agromyzidae, in eDNA samples.

Figure S2. The standardized (rarefied) results of sequence reads that were detected from samples collected from the surface of field cabbage by tap water or rainfall.

Table S1. Number of reads that remained in data pre-processing and post-processing

Table S2. **Table S2_rev2.xlsx**: List of sequences and taxonomic information of each ASV.

Table S3. Sum of sequence reads of all the same lowest taxonomic groups at least family level.

Table S4. The list of species detected from surface of cabbage having chewing damage, and their sequence reads.

Table S5. Primers of the first PCR

Table S6. Primer for 2^nd^ PCR

Table S7. **Table_S7_rev2.xlsx**: List of indexes and reads of each sample by each ASV.

Table S8. **iNEXT_info_yoneya_SI_rev2.xlsx**

Table S9. **iNEXT_info_yoneya_Fig01_rev2.xlsx**

Table S10. **iNEXT_info_yoneya_eggplant_rev2.xlsx**

**Coverage-based methods and results**

When we applied the coverage-based method, iNEXT:: DataInfo() function, to all the samples except for negative controls, the minimum coverage was relatively low (60.66 % of S05) (Table S8). Since rarefying the samples with such a low coverage loses too many sequences, we rarefied the data with the minimum coverage among focal samples only.

First, when comparing the differences in detecting performance between tap water and rainfall for the field samples (see Fig.1), the minimum coverage was 98.467% (Table S9) and we rarefied the data with this coverage, resulting in Fig. S2. With this common coverage (98.467%), we also compared the ASV richness between samples with tap water (n = 2) and those with rainfall (n = 3). The average of the estimated ASV richness with the standardized sample coverage (98.5 %) was significantly greater in the rainfall samples (linear model, P = 0.000527). The 95% confidence interval was [-29.29117, 247.0779] and [289.8204, 362.0253] for the tap water and rain fall, respectively.

Second, when comparing the differences between tap water (n = 12) and distilled water (n = 2) with eggplants samples (see “**Pros and cons for using three types of water for plant flow method**” subsection in the main text), the minimum coverage was 92.3% (Table S10) and we compared the ASV richness. The average of the estimated ASV richness with the standardized sample coverage (92.3 %) was not statistically different (linear model, P = 0.747). The 95% confidence interval was [3.495311, 17.40478]) and [-13.44648, 29.03981] for the tap water and distilled water, respectively.


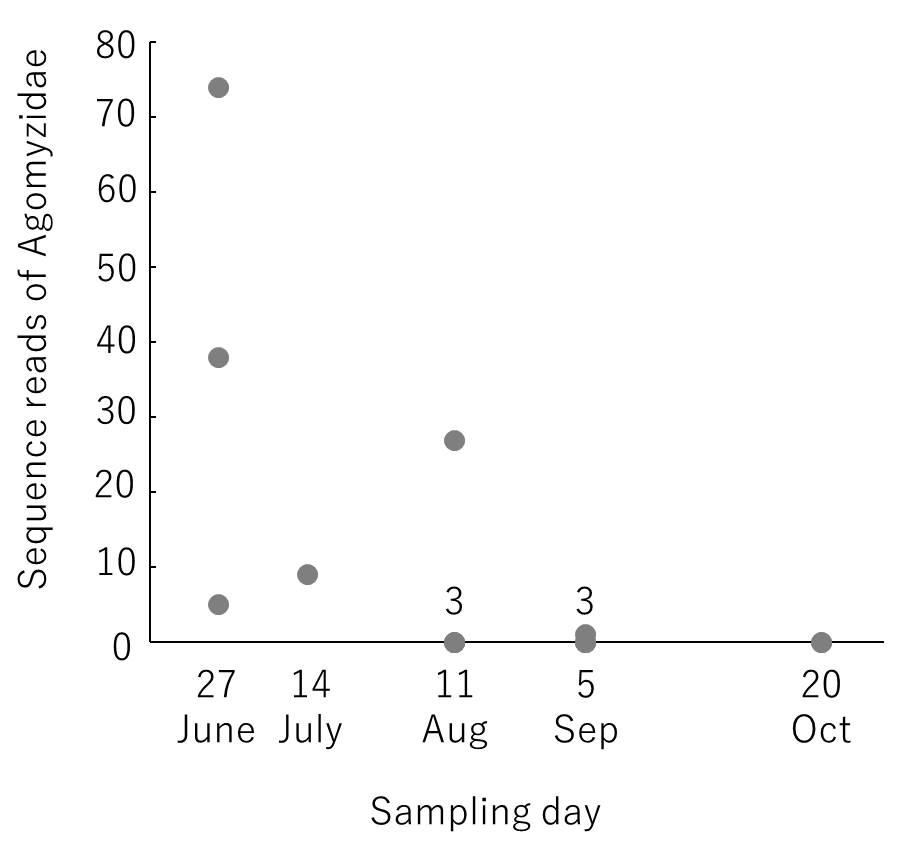


**Figure S1.** The total sequence reads of leaf-miner flies, Agromyzidae, in eDNA samples. The eDNA samples were collected from egg plants surface by the “plant flow collection” method at each sampling day in 2017. N = 3 (27 June, S1-3), 4 (11 August, S28-31), 4 (5 September, S32-35), 2 (20 October, S49,50). The numbers above plots are the number of samples showing the same 0 value.


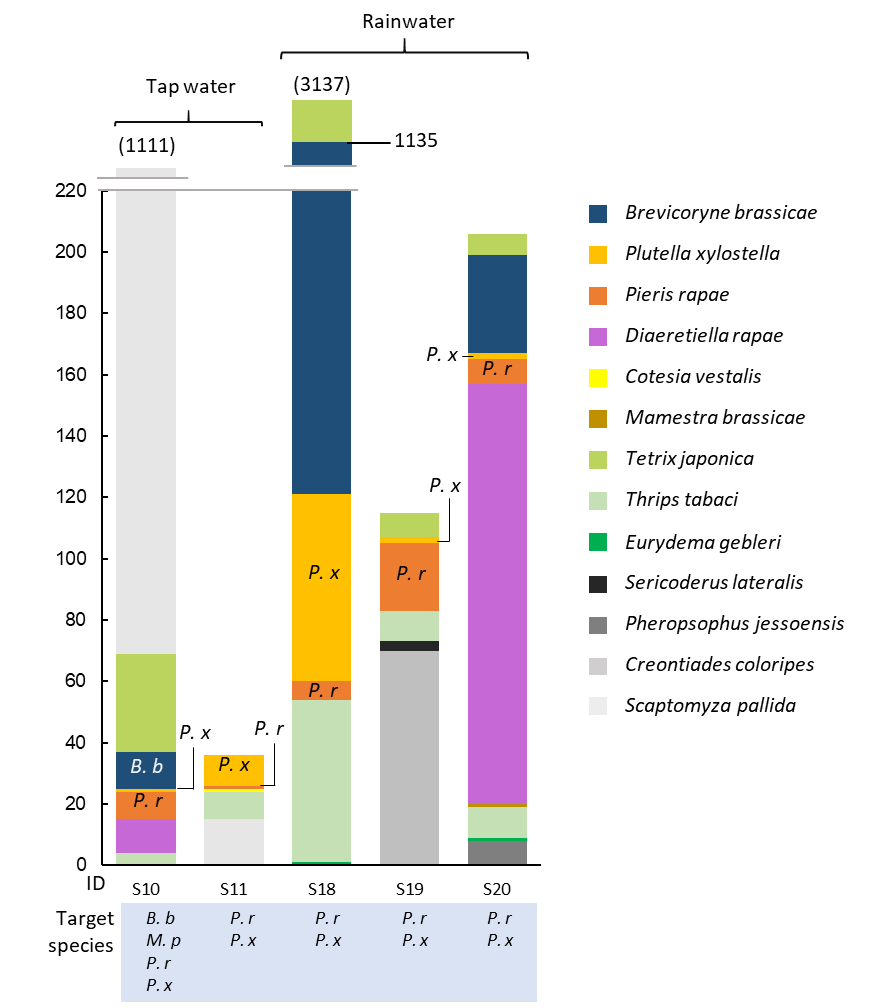


**Figure S2.** The standardized (rarefied) results of sequence reads, which were detected from samples collected from the surface of field cabbage by tap water (S10 and S11) or rainfall (S18-20). The sequence data processing was conducted with a modified setting and standardized the sequencing efforts by the “coverage-based” method. Label of under each bar is sample ID and the target species of each sample, *B. b*: *Brevicoryne brassicae*, *M. p*: *Myzus persicael*, *P. r*; *Pieris rapae*, *P.x*: *Plutella xylostella*. Target species were visually observed on a cabbage plant at the sampling day on July 4 (S10 and S11) and July 7, 2017 (S18-20). When target species were detected in a sample, the abbreviation of species name is described in a graph. The value in the parenthesis is the total sequence reads of S18. The number of target species observed on a sampling plant is described in Table 4.

**Table S1** Number of reads that remained in data pre-processing and post-processing

|  |  | **Pre-processing reads and prop.** | | | | | | | | |  | **Post-processing reads and prop.** | | | |
| --- | --- | --- | --- | --- | --- | --- | --- | --- | --- | --- | --- | --- | --- | --- | --- |
| **Sample ID** | **Sample attribute** | **Original FASTQ (reads)** | **Primer removed (reads)** | **Input (reads)** | **Quality filtered (reads)** | **Denoised (reads)** | **Pair-reads merged (reads)** | **Chimera removed (reads)** | **Nonchimera /Input (prop.)** | **Nonchimera /Original (prop.)** |  | **Delete**  **negative control** | **arthropoda** | **family** | **species** |
| S01 | Eggplant_aphid_tap_01 | 1,543 | 1,059 | 1,059 | 990 | 988 | 961 | 961 | 0.907 | 0.623 |  | 891 | 834 | 834 | 776 |
| S02 | Eggplant_aphid_tap_02 | 163 | 150 | 150 | 138 | 138 | 129 | 129 | 0.860 | 0.791 |  | 103 | 89 | 89 | 82 |
| S03 | Eggplant_aphid_tap_03 | 6,981 | 883 | 883 | 547 | 544 | 542 | 541 | 0.613 | 0.077 |  | 481 | 444 | 443 | 357 |
| S04 | cabbage_chewing-damage_tap_01 | 172 | 86 | 86 | 71 | 71 | 70 | 70 | 0.814 | 0.407 |  | 54 | 17 | 15 | 14 |
| S05 | cabbage_cabbage-aphid_tap_01 | 1,791 | 206 | 206 | 108 | 104 | 97 | 97 | 0.471 | 0.054 |  | 25 | 7 | 5 | 5 |
| S06 | cabbage_cabbage-aphid_tap_02 | 416 | 180 | 180 | 140 | 138 | 131 | 131 | 0.728 | 0.315 |  | 95 | 32 | 26 | 24 |
| S07 | cabbage_white-butterfly_tap_01 | 60 | 59 | 59 | 59 | 59 | 59 | 59 | 1 | 0.983 |  | 55 | 55 | 55 | 55 |
| S08 | cabbage_green-peach-aphid_tap_01 | 42,206 | 41,614 | 41,614 | 41,183 | 41,180 | 41,136 | 41,117 | 0.988 | 0.974 |  | 41,058 | 40,893 | 40,893 | 40,893 |
| S09 | cabbage_white-butterfly_tap_02 | 3,671 | 3,189 | 3,189 | 3,048 | 3,045 | 3,018 | 3,018 | 0.946 | 0.822 |  | 2,931 | 2,869 | 2,868 | 2,865 |
| S10 | cabbage_field_tap_01 | 3,490 | 3,168 | 3,168 | 3,100 | 3,091 | 3,062 | 3,059 | 0.966 | 0.877 |  | 3,035 | 1,423 | 1,137 | 1,117 |
| S11 | cabbage_field_tap_02 | 107,585 | 106,352 | 106,352 | 104,357 | 103,698 | 103,494 | 103,433 | 0.973 | 0.961 |  | 103,346 | 47,742 | 45,298 | 1,629 |
| S12 | cabbage_chewing-damage_tap_02 | 2,238 | 2,042 | 2,042 | 1,972 | 1,959 | 1,898 | 1,898 | 0.929 | 0.848 |  | 1,696 | 224 | 210 | 174 |
| S13 | cabbage_cabbage-aphid_tap_03 | 28,331 | 26,433 | 26,433 | 26,095 | 26,093 | 26,053 | 26,052 | 0.986 | 0.920 |  | 25,939 | 25,859 | 25,859 | 25,680 |
| S14 | cabbage_cabbage-aphid_tap_04 | 1,541 | 268 | 268 | 133 | 131 | 131 | 131 | 0.489 | 0.085 |  | 42 | 17 | 14 | 14 |
| S15 | cabbage_white-butterfly_tap_03 | 1,864 | 258 | 258 | 148 | 145 | 142 | 142 | 0.550 | 0.076 |  | 26 | 1 | 1 | 0 |
| S16 | cabbage_green-peach-aphid_tap_03 | 1,609 | 335 | 335 | 185 | 185 | 161 | 161 | 0.481 | 0.100 |  | 53 | 5 | 5 | 3 |
| S17 | cabbage_white-butterfly_tap_04 | 2,194 | 1,697 | 1,697 | 1,630 | 1,619 | 1,570 | 1,568 | 0.924 | 0.715 |  | 1,385 | 1,311 | 1,306 | 1,278 |
| S18 | cabbage_field_rain_01 | 211,475 | 205,156 | 205,156 | 202,136 | 201,833 | 201,272 | 200,910 | 0.979 | 0.950 |  | 200,862 | 87,266 | 79,106 | 78,661 |
| S19 | cabbage_field_rain_02 | 292,539 | 290,511 | 290,511 | 285,865 | 285,470 | 283,961 | 281,902 | 0.970 | 0.964 |  | 281,846 | 69,351 | 3,551 | 3,410 |
| S20 | cabbage_field_rain_03 | 152,676 | 151,412 | 151,412 | 148,001 | 147,809 | 147,488 | 146,996 | 0.971 | 0.963 |  | 146,901 | 22,347 | 17,282 | 3,822 |
| S21 | cabbage_chewing-damage_tap_03 | 7,130 | 5,057 | 5,057 | 4,814 | 4,807 | 4,778 | 4,778 | 0.945 | 0.670 |  | 4,540 | 5 | 5 | 5 |
| S22 | cabbage_cabbage-aphid_tap_05 | 10,443 | 10,162 | 10,162 | 10,004 | 10,004 | 9,999 | 9,999 | 0.984 | 0.957 |  | 9,900 | 9,591 | 9,591 | 9,585 |
| S23 | cabbage_cabbage-aphid_tap_06 | 2,605 | 2,317 | 2,317 | 2,229 | 2,226 | 2,219 | 2,219 | 0.958 | 0.852 |  | 2,108 | 1,418 | 1,414 | 1,410 |
| S24 | cabbage_ green-peach-aphid_tap_04 | 1,711 | 1,274 | 1,274 | 1,209 | 1,207 | 1,204 | 1,204 | 0.945 | 0.704 |  | 1,104 | 26 | 23 | 23 |
| S25 | cabbage_ white-butterfly_tap_05 | 1,060 | 604 | 604 | 567 | 561 | 557 | 557 | 0.922 | 0.525 |  | 461 | 65 | 65 | 31 |
| S26 | cabbage_white-butterfly_tap_06 | 1,319 | 1,190 | 1,190 | 1,160 | 1,157 | 1,149 | 1,146 | 0.963 | 0.869 |  | 1,056 | 338 | 316 | 298 |
| S27 | eggplant_aphid_tap_04 | 11,845 | 10,578 | 10,578 | 10,319 | 10,292 | 10,260 | 9,331 | 0.882 | 0.788 |  | 9,159 | 1,198 | 960 | 642 |
| S28 | eggplant_leafminer_tap_01 | 7,984 | 7,634 | 7,634 | 7,502 | 7,471 | 7,451 | 7,447 | 0.976 | 0.933 |  | 7,288 | 148 | 76 | 12 |
| S29 | eggplant_leafminer_tap_02 | 12,038 | 11,689 | 11,689 | 11,544 | 11,501 | 11,475 | 11,472 | 0.981 | 0.953 |  | 11,297 | 55 | 29 | 7 |
| S30 | eggplant_leafminer_tap_03 | 19,432 | 17,762 | 17,762 | 17,459 | 17,416 | 17,396 | 17,393 | 0.979 | 0.895 |  | 17,221 | 69 | 69 | 62 |
| S31 | eggplant_leafminer_tap_04 | 33,065 | 32,065 | 32,065 | 31,676 | 31,622 | 31,591 | 31,588 | 0.985 | 0.955 |  | 31,396 | 32 | 29 | 24 |
| S32 | eggplant_leafminer_tap_05 | 5,190 | 4,451 | 4,451 | 4,351 | 4,344 | 4,329 | 4,329 | 0.973 | 0.834 |  | 4,207 | 4,164 | 4,163 | 0 |
| S33 | eggplant_leafminer_tap_06 | 1,776 | 1,615 | 1,615 | 1,581 | 1,576 | 1,571 | 1,571 | 0.973 | 0.885 |  | 1,513 | 1,429 | 1,427 | 26 |
| S34 | eggplant_leafminer_tap_07 | 1,670 | 928 | 928 | 866 | 864 | 859 | 858 | 0.925 | 0.514 |  | 795 | 773 | 769 | 13 |
| S35 | eggplant_leafminer_tap_08 | 2,048 | 883 | 883 | 777 | 776 | 769 | 758 | 0.858 | 0.370 |  | 664 | 478 | 473 | 13 |
| S44 | Negative_control_air_01 | 236 | 36 | 36 | 16 | 16 | 16 | 16 | 0.444 | 0.068 |  | - | - | - | - |
| S45 | Negative_control_air_02 | 2129 | 333 | 333 | 150 | 150 | 150 | 150 | 0.450 | 0.070 |  | - | - | - | - |
| S46 | Negative_control_air_03 | 133 | 17 | 17 | 6 | 6 | 5 | 5 | 0.294 | 0.038 |  | - | - | - | - |
| S47 | Negative_control_air_04 | 672 | 62 | 62 | 29 | 29 | 28 | 28 | 0.452 | 0.042 |  | - | - | - | - |
| S48 | Negative_control_air_05 | 136 | 31 | 31 | 22 | 21 | 18 | 17 | 0.548 | 0.125 |  | - | - | - | - |
| S49 | eggplant_aphid_DW_01 | 1,585 | 1,148 | 1,148 | 1,091 | 1,086 | 1,072 | 1,061 | 0.924 | 0.669 |  | 1,007 | 873 | 873 | 396 |
| S50 | eggplant_aphid_DW_02 | 470 | 227 | 227 | 203 | 202 | 195 | 193 | 0.850 | 0.411 |  | 170 | 131 | 131 | 19 |
| S55 | negative-control_01 | 1,643 | 420 | 420 | 276 | 273 | 258 | 258 | 0.614 | 0.157 |  | - | - | - | - |
| S56 | negative-control_02 | 7,797 | 6,959 | 6,959 | 6,200 | 6,196 | 5,075 | 5,075 | 0.729 | 0.651 |  | - | - | - | - |
|  |  |  |  |  |  |  |  |  |  |  |  |  |  |  |  |
| Total |  | 996,662 | 952,500 | 933,957 | 932,103 | 927,805 | 927,805 | 923,828 |  |  |  | 914,710 | 321,579 | 239,410 | 173,425 |
| Average |  | 22,651 | 21,648 | 21,226 | 21,184 | 21,086 | 21,086 | 20,996 |  |  |  | 24,722 | 8,691 | 6,471 | 4,687 |
| S.E. |  | 8,826 | 8,737 | 8,594 | 8,580 | 8,547 | 8,547 | 8,506 |  |  |  | 10,011 | 3,342 | 2,676 | 2,426 |

**Table S3.** Sum of sequence reads of all the same lowest taxonomic groups at least family level.

| class | order | family | genus | species | S01 | S02 | S03 | S04 | S05 | S06 | S07 | S08 | S09 | S10 | S11 | S12 | S13 | S14 | S15 | S16 | S17 | S18 | S19 | S20 | S21 | S22 | S23 | S24 | S25 | S26 | S27 | S28 | S29 | S30 | S31 | S32 | S33 | S34 | S35 | S49 | S50 |
| --- | --- | --- | --- | --- | --- | --- | --- | --- | --- | --- | --- | --- | --- | --- | --- | --- | --- | --- | --- | --- | --- | --- | --- | --- | --- | --- | --- | --- | --- | --- | --- | --- | --- | --- | --- | --- | --- | --- | --- | --- | --- |
| Arachnida | |  |  |  |  |  |  |  |  |  |  |  |  |  |  |  |  |  |  |  |  |  |  |  |  |  |  |  |  |  |  |  |  |  |  |  |  |  |  |  |  |
|  | Araneae | Tetragnathidae | *Leucauge* |  | 0 | 0 | 0 | 0 | 0 | 0 | 0 | 0 | 0 | 0 | 0 | 0 | 0 | 0 | 0 | 0 | 0 | 0 | 0 | 14 | 0 | 0 | 0 | 0 | 0 | 0 | 0 | 0 | 0 | 0 | 0 | 0 | 0 | 0 | 0 | 0 | 0 |
|  | Mesostigmata | Phytoseiidae | *Euseius* |  | 0 | 0 | 0 | 0 | 0 | 0 | 0 | 0 | 0 | 0 | 0 | 0 | 0 | 0 | 0 | 0 | 0 | 6 | 0 | 0 | 0 | 0 | 0 | 0 | 0 | 0 | 0 | 0 | 0 | 0 | 0 | 0 | 0 | 0 | 0 | 0 | 0 |
|  | Mesostigmata | Phytoseiidae |  |  | 0 | 0 | 0 | 0 | 0 | 0 | 0 | 0 | 0 | 0 | 0 | 0 | 0 | 0 | 0 | 0 | 0 | 0 | 0 | 9 | 0 | 0 | 0 | 0 | 0 | 0 | 0 | 0 | 0 | 0 | 0 | 0 | 0 | 0 | 0 | 0 | 0 |
|  | Sarcoptiformes | Acaridae | *Tyrophagus* | *T. putrescentiae* | 0 | 0 | 0 | 0 | 0 | 0 | 0 | 0 | 0 | 0 | 0 | 0 | 0 | 0 | 0 | 0 | 0 | 0 | 0 | 0 | 0 | 0 | 0 | 0 | 0 | 0 | 0 | 0 | 0 | 0 | 21 | 0 | 0 | 0 | 0 | 0 | 0 |
|  | Sarcoptiformes | Acaridae |  |  | 0 | 0 | 0 | 0 | 0 | 0 | 0 | 0 | 0 | 0 | 0 | 0 | 0 | 0 | 0 | 0 | 0 | 0 | 0 | 95 | 0 | 0 | 0 | 0 | 0 | 0 | 0 | 0 | 0 | 0 | 0 | 0 | 0 | 0 | 0 | 0 | 0 |
|  | Trombidiformes | Eriophyidae |  |  | 0 | 0 | 0 | 0 | 0 | 0 | 0 | 0 | 0 | 0 | 0 | 0 | 0 | 0 | 0 | 0 | 0 | 0 | 0 | 10 | 0 | 0 | 0 | 0 | 0 | 0 | 0 | 0 | 0 | 0 | 0 | 0 | 0 | 0 | 0 | 0 | 0 |
|  | Trombidiformes | Eupodidae |  |  | 0 | 0 | 0 | 0 | 0 | 0 | 0 | 0 | 0 | 0 | 0 | 0 | 0 | 0 | 0 | 0 | 0 | 0 | 54 | 33 | 0 | 0 | 0 | 0 | 0 | 0 | 0 | 0 | 0 | 0 | 0 | 0 | 0 | 0 | 0 | 0 | 0 |
|  | Trombidiformes | Tarsonemidae |  |  | 1 | 0 | 5 | 0 | 0 | 0 | 0 | 0 | 0 | 0 | 0 | 0 | 0 | 0 | 0 | 0 | 6 | 0 | 0 | 0 | 0 | 1 | 0 | 0 | 0 | 1 | 52 | 0 | 0 | 0 | 0 | 0 | 0 | 0 | 4 | 116 | 80 |
|  | Trombidiformes | Tenuipalpidae | *Brevipalpus* |  | 0 | 0 | 0 | 0 | 0 | 0 | 0 | 0 | 0 | 0 | 0 | 0 | 1 | 0 | 0 | 0 | 0 | 0 | 0 | 0 | 0 | 0 | 0 | 0 | 0 | 0 | 0 | 0 | 0 | 0 | 0 | 0 | 0 | 3 | 0 | 1 | 0 |
|  | Trombidiformes | Tetranychidae | *Tetranychus* |  | 40 | 1 | 0 | 0 | 0 | 0 | 0 | 0 | 0 | 0 | 0 | 0 | 0 | 0 | 0 | 0 | 0 | 0 | 0 | 0 | 0 | 0 | 0 | 0 | 35 | 15 | 242 | 5 | 23 | 0 | 3 | 4159 | 1401 | 754 | 451 | 22 | 0 |
| Collembola | |  |  |  |  |  |  |  |  |  |  |  |  |  |  |  |  |  |  |  |  |  |  |  |  |  |  |  |  |  |  |  |  |  |  |  |  |  |  |  |  |
|  | Entomobryomorpha | Entomobryidae | *Entomobrya* |  | 0 | 0 | 0 | 0 | 0 | 0 | 0 | 0 | 0 | 14 | 10 | 0 | 0 | 0 | 0 | 0 | 0 | 0 | 10 | 0 | 0 | 0 | 0 | 0 | 0 | 0 | 0 | 0 | 0 | 0 | 0 | 0 | 0 | 0 | 0 | 0 | 0 |
|  | Entomobryomorpha | Entomobryidae |  |  | 0 | 0 | 0 | 0 | 0 | 0 | 0 | 0 | 0 | 0 | 64 | 0 | 0 | 0 | 0 | 0 | 0 | 0 | 0 | 0 | 0 | 0 | 0 | 0 | 0 | 0 | 0 | 0 | 0 | 0 | 0 | 0 | 0 | 0 | 0 | 0 | 0 |
|  | Entomobryomorpha | Isotomidae | *Desoria* |  | 0 | 0 | 0 | 0 | 0 | 0 | 0 | 0 | 0 | 0 | 0 | 5 | 0 | 0 | 0 | 0 | 0 | 0 | 8 | 11047 | 0 | 1 | 1 | 0 | 0 | 0 | 0 | 55 | 0 | 0 | 0 | 0 | 0 | 0 | 0 | 0 | 0 |
|  | Entomobryomorpha | Isotomidae |  |  | 0 | 0 | 0 | 0 | 0 | 0 | 0 | 0 | 0 | 0 | 0 | 0 | 0 | 0 | 0 | 0 | 0 | 0 | 0 | 316 | 0 | 0 | 0 | 0 | 0 | 0 | 0 | 0 | 0 | 0 | 0 | 0 | 0 | 0 | 0 | 0 | 0 |
|  | Symphypleona | Bourletiellidae |  |  | 0 | 0 | 0 | 0 | 0 | 0 | 0 | 0 | 0 | 0 | 29 | 0 | 0 | 0 | 0 | 0 | 0 | 8 | 0 | 70 | 0 | 0 | 0 | 0 | 0 | 0 | 0 | 1 | 0 | 0 | 0 | 0 | 0 | 0 | 0 | 0 | 0 |
|  | Symphypleona | Katiannidae | *Sminthurinus* |  | 0 | 2 | 0 | 1 | 0 | 0 | 0 | 0 | 0 | 0 | 0 | 0 | 1 | 0 | 0 | 0 | 19 | 0 | 0 | 0 | 0 | 0 | 0 | 0 | 0 | 0 | 1 | 0 | 0 | 7 | 0 | 0 | 0 | 0 | 0 | 0 | 0 |
| Insecta | |  |  |  |  |  |  |  |  |  |  |  |  |  |  |  |  |  |  |  |  |  |  |  |  |  |  |  |  |  |  |  |  |  |  |  |  |  |  |  |  |
|  | Coleoptera | Carabidae | *Harpalus* |  | 0 | 0 | 0 | 0 | 0 | 0 | 0 | 0 | 0 | 0 | 0 | 0 | 0 | 0 | 0 | 0 | 0 | 0 | 0 | 33 | 0 | 0 | 0 | 0 | 0 | 0 | 0 | 0 | 0 | 0 | 0 | 0 | 0 | 0 | 0 | 0 | 0 |
|  | Coleoptera | Carabidae | *Pheropsophus* | *P. jessoensis* | 0 | 0 | 0 | 0 | 0 | 0 | 0 | 0 | 0 | 0 | 0 | 0 | 0 | 0 | 0 | 0 | 0 | 0 | 0 | 104 | 0 | 0 | 0 | 0 | 0 | 0 | 0 | 1 | 0 | 0 | 0 | 0 | 0 | 0 | 0 | 0 | 0 |
|  | Coleoptera | Corylophidae | *Sericoderus* | *S. lateralis* | 0 | 0 | 0 | 0 | 0 | 0 | 0 | 0 | 0 | 0 | 0 | 0 | 0 | 0 | 0 | 0 | 0 | 0 | 82 | 0 | 0 | 0 | 0 | 0 | 0 | 0 | 0 | 0 | 0 | 0 | 0 | 0 | 0 | 0 | 0 | 0 | 0 |
|  | Diptera | Agromyzidae | *Liriomyza* | *L. sativae* | 74 | 5 | 38 | 0 | 0 | 0 | 0 | 0 | 129 | 0 | 0 | 17 | 0 | 0 | 0 | 2 | 0 | 0 | 0 | 0 | 0 | 0 | 0 | 0 | 1 | 0 | 9 | 0 | 0 | 27 | 0 | 0 | 0 | 0 | 1 | 0 | 0 |
|  | Diptera | Agromyzidae |  |  | 8 | 0 | 1 | 0 | 0 | 0 | 0 | 0 | 0 | 0 | 0 | 0 | 0 | 0 | 0 | 0 | 0 | 0 | 0 | 0 | 0 | 0 | 0 | 0 | 0 | 0 | 0 | 0 | 0 | 0 | 0 | 0 | 0 | 0 | 0 | 0 | 0 |
|  | Diptera | Cecidomyiidae |  |  | 0 | 0 | 0 | 0 | 0 | 0 | 0 | 0 | 0 | 0 | 0 | 0 | 0 | 0 | 0 | 0 | 1 | 4 | 0 | 0 | 0 | 0 | 0 | 0 | 0 | 0 | 0 | 0 | 0 | 0 | 0 | 0 | 0 | 0 | 0 | 0 | 0 |
|  | Diptera | Chironomidae | *Cladotanytarsus* | *C. vanderwulpi* | 0 | 0 | 0 | 0 | 0 | 0 | 0 | 4 | 0 | 0 | 0 | 0 | 0 | 0 | 0 | 0 | 0 | 0 | 0 | 0 | 0 | 0 | 0 | 0 | 0 | 0 | 0 | 0 | 0 | 0 | 0 | 0 | 0 | 0 | 0 | 0 | 0 |
|  | Diptera | Culicidae | *Aedes* | *A. albopictus* | 0 | 0 | 0 | 0 | 0 | 0 | 0 | 0 | 0 | 0 | 0 | 0 | 0 | 0 | 0 | 0 | 0 | 0 | 0 | 0 | 0 | 0 | 0 | 0 | 0 | 0 | 0 | 0 | 0 | 0 | 0 | 0 | 2 | 0 | 0 | 0 | 0 |
|  | Diptera | Drosophilidae | *Drosophila* |  | 0 | 0 | 0 | 0 | 0 | 0 | 0 | 0 | 0 | 0 | 0 | 0 | 0 | 0 | 0 | 0 | 0 | 11 | 0 | 0 | 0 | 0 | 0 | 0 | 0 | 0 | 0 | 0 | 0 | 0 | 0 | 0 | 0 | 0 | 0 | 0 | 0 |
|  | Diptera | Drosophilidae | *Scaptomyza* | *S. pallida* | 0 | 0 | 0 | 0 | 1 | 0 | 0 | 0 | 0 | 1045 | 667 | 0 | 0 | 0 | 0 | 0 | 0 | 6 | 0 | 0 | 0 | 0 | 0 | 0 | 0 | 0 | 0 | 0 | 0 | 0 | 0 | 0 | 0 | 0 | 0 | 0 | 0 |
|  | Diptera | Drosophilidae | *Scaptomyza* |  | 0 | 0 | 1 | 0 | 0 | 0 | 0 | 0 | 0 | 3 | 43481 | 0 | 0 | 0 | 0 | 0 | 0 | 86 | 45 | 53 | 0 | 0 | 0 | 0 | 0 | 2 | 1 | 0 | 0 | 0 | 0 | 0 | 0 | 0 | 6 | 0 | 0 |
|  | Diptera | Psychodidae | *Psychoda* |  | 0 | 0 | 0 | 0 | 0 | 0 | 0 | 0 | 0 | 0 | 9 | 0 | 0 | 0 | 1 | 0 | 0 | 0 | 0 | 31 | 0 | 0 | 0 | 0 | 0 | 0 | 0 | 0 | 0 | 0 | 0 | 0 | 0 | 0 | 0 | 0 | 0 |
|  | Diptera | Syrphidae | *Sphaerophoria* | *S. macrogaster* | 0 | 0 | 0 | 0 | 0 | 18 | 0 | 0 | 0 | 0 | 0 | 0 | 0 | 0 | 0 | 0 | 0 | 0 | 0 | 0 | 0 | 0 | 0 | 0 | 0 | 0 | 0 | 0 | 0 | 0 | 0 | 0 | 0 | 0 | 0 | 0 | 0 |
|  | Hemiptera | Aleyrodidae | *Trialeurodes* | *T. vaporariorum* | 0 | 0 | 0 | 0 | 0 | 0 | 0 | 0 | 0 | 0 | 0 | 0 | 0 | 0 | 0 | 0 | 0 | 0 | 0 | 0 | 0 | 0 | 0 | 0 | 0 | 0 | 0 | 0 | 7 | 0 | 0 | 0 | 0 | 0 | 0 | 0 | 0 |
|  | Hemiptera | Aphididae | *Aphis* | *A. gossypii* | 143 | 38 | 179 | 0 | 0 | 1 | 0 | 0 | 0 | 0 | 0 | 0 | 0 | 0 | 0 | 0 | 0 | 0 | 0 | 0 | 0 | 0 | 0 | 0 | 0 | 0 | 22 | 0 | 0 | 0 | 0 | 0 | 0 | 0 | 0 | 305 | 19 |
|  | Hemiptera | Aphididae | *Aphis* |  | 10 | 5 | 79 | 0 | 0 | 0 | 0 | 0 | 0 | 0 | 0 | 0 | 0 | 0 | 0 | 0 | 0 | 0 | 0 | 0 | 0 | 0 | 0 | 0 | 0 | 0 | 23 | 0 | 0 | 0 | 0 | 0 | 0 | 0 | 0 | 339 | 32 |
|  | Hemiptera | Aphididae | *Brevicoryne* | *B. brassicae* | 0 | 0 | 0 | 0 | 0 | 0 | 0 | 0 | 0 | 13 | 0 | 0 | 19907 | 2 | 0 | 0 | 2 | 24905 | 6 | 629 | 3 | 254 | 362 | 0 | 0 | 105 | 2 | 0 | 0 | 0 | 0 | 0 | 0 | 1 | 0 | 0 | 0 |
|  | Hemiptera | Aphididae | *Myzus* | *M. persicae* | 0 | 1 | 4 | 0 | 1 | 1 | 55 | 40886 | 0 | 0 | 0 | 0 | 0 | 0 | 0 | 1 | 0 | 0 | 10 | 0 | 0 | 0 | 0 | 17 | 0 | 0 | 2 | 0 | 0 | 0 | 0 | 0 | 0 | 0 | 0 | 0 | 0 |
|  | Hemiptera | Aphididae |  |  | 0 | 0 | 0 | 0 | 0 | 0 | 0 | 0 | 0 | 0 | 0 | 0 | 177 | 0 | 0 | 0 | 0 | 259 | 0 | 0 | 0 | 4 | 3 | 0 | 0 | 1 | 0 | 0 | 0 | 0 | 0 | 0 | 0 | 0 | 0 | 0 | 0 |
|  | Hemiptera | Miridae | *Creontiades* | *C. coloripes* | 0 | 0 | 0 | 0 | 0 | 0 | 0 | 0 | 0 | 0 | 2 | 0 | 0 | 0 | 0 | 0 | 0 | 0 | 2143 | 0 | 0 | 0 | 0 | 0 | 0 | 0 | 12 | 0 | 0 | 0 | 0 | 0 | 0 | 0 | 0 | 0 | 0 |
|  | Hemiptera | Pemphigidae | *Tetraneura* |  | 0 | 0 | 0 | 0 | 0 | 0 | 0 | 0 | 0 | 0 | 0 | 0 | 0 | 0 | 0 | 0 | 0 | 0 | 0 | 1201 | 0 | 0 | 0 | 0 | 0 | 0 | 0 | 4 | 0 | 0 | 0 | 0 | 0 | 0 | 0 | 0 | 0 |
|  | Hemiptera | Pentatomidae | *Eurydema* | *E. gebleri* | 0 | 0 | 0 | 0 | 0 | 0 | 0 | 0 | 0 | 0 | 20 | 0 | 0 | 0 | 0 | 0 | 0 | 9 | 18 | 5 | 0 | 0 | 0 | 0 | 0 | 0 | 0 | 0 | 0 | 0 | 0 | 0 | 0 | 0 | 0 | 0 | 0 |
|  | Hemiptera | Pseudococcidae | *Planococcus* | *P. citri* | 0 | 0 | 0 | 0 | 0 | 0 | 0 | 0 | 68 | 0 | 0 | 0 | 0 | 0 | 0 | 0 | 3 | 0 | 0 | 0 | 0 | 0 | 0 | 0 | 0 | 0 | 0 | 6 | 0 | 0 | 3 | 0 | 0 | 0 | 0 | 0 | 0 |
|  | Hymenoptera | Braconidae | *Aphidius* | *A. colemani* | 0 | 0 | 0 | 0 | 0 | 0 | 0 | 0 | 0 | 0 | 0 | 0 | 8 | 3 | 0 | 0 | 0 | 0 | 0 | 0 | 0 | 0 | 0 | 6 | 0 | 0 | 0 | 0 | 0 | 0 | 0 | 0 | 0 | 0 | 0 | 0 | 0 |
|  | Hymenoptera | Braconidae | *Cotesia* | *C. vestalis* | 0 | 0 | 0 | 0 | 0 | 0 | 0 | 0 | 0 | 0 | 17 | 0 | 0 | 0 | 0 | 0 | 0 | 0 | 0 | 0 | 0 | 0 | 0 | 0 | 0 | 0 | 0 | 0 | 0 | 0 | 0 | 0 | 0 | 0 | 0 | 0 | 0 |
|  | Hymenoptera | Braconidae | *Cotesia* |  | 0 | 0 | 0 | 0 | 0 | 0 | 0 | 0 | 0 | 0 | 76 | 0 | 0 | 0 | 0 | 0 | 0 | 0 | 23 | 38 | 0 | 0 | 0 | 0 | 0 | 0 | 0 | 0 | 0 | 0 | 0 | 0 | 0 | 0 | 0 | 0 | 0 |
|  | Hymenoptera | Braconidae | *Diaeretiella* | *D. rapae* | 0 | 0 | 0 | 0 | 0 | 0 | 0 | 0 | 0 | 11 | 0 | 1 | 4441 | 1 | 0 | 0 | 0 | 0 | 0 | 2599 | 0 | ### | 896 | 0 | 0 | 0 | 0 | 3 | 0 | 35 | 0 | 0 | 0 | 0 | 0 | 0 | 0 |
|  | Hymenoptera | Eulophidae | *Neochrysocharis* |  | 0 | 0 | 0 | 0 | 0 | 0 | 0 | 0 | 0 | 0 | 0 | 0 | 0 | 0 | 0 | 2 | 2 | 0 | 0 | 0 | 0 | 0 | 0 | 0 | 0 | 0 | 0 | 0 | 0 | 0 | 0 | 0 | 0 | 0 | 0 | 0 | 0 |
|  | Hymenoptera | Figitidae | *Kleidotoma* |  | 0 | 0 | 0 | 0 | 0 | 0 | 0 | 0 | 0 | 3 | 0 | 0 | 0 | 0 | 0 | 0 | 0 | 0 | 0 | 0 | 0 | 0 | 0 | 0 | 0 | 0 | 0 | 0 | 0 | 0 | 0 | 0 | 0 | 0 | 0 | 0 | 0 |
|  | Hymenoptera | Figitidae |  |  | 0 | 0 | 0 | 0 | 0 | 0 | 0 | 0 | 0 | 0 | 0 | 0 | 0 | 0 | 0 | 0 | 0 | 43 | 0 | 0 | 0 | 0 | 0 | 0 | 0 | 0 | 0 | 0 | 0 | 0 | 0 | 0 | 0 | 0 | 0 | 0 | 0 |
|  | Hymenoptera | Pteromalidae |  |  | 0 | 0 | 0 | 0 | 0 | 0 | 0 | 0 | 0 | 0 | 0 | 0 | 0 | 0 | 0 | 0 | 0 | 0 | 0 | 3 | 0 | 0 | 0 | 0 | 0 | 0 | 0 | 0 | 0 | 0 | 0 | 0 | 0 | 0 | 0 | 0 | 0 |
|  | Hymenoptera | Trichogrammatidae | *Trichogramma* |  | 0 | 0 | 0 | 0 | 0 | 0 | 0 | 0 | 0 | 0 | 0 | 0 | 0 | 0 | 0 | 0 | 0 | 6 | 0 | 0 | 0 | 0 | 0 | 0 | 0 | 0 | 0 | 0 | 0 | 0 | 0 | 0 | 0 | 0 | 0 | 0 | 0 |
|  | Lepidoptera | Crambidae | *Bradina* | *B. diagonalis* | 0 | 0 | 0 | 0 | 0 | 0 | 0 | 0 | 0 | 0 | 0 | 0 | 0 | 0 | 0 | 0 | 0 | 0 | 0 | 2 | 0 | 0 | 0 | 0 | 0 | 0 | 0 | 0 | 0 | 0 | 0 | 0 | 0 | 0 | 0 | 0 | 0 |
|  | Lepidoptera | Crambidae | *Udea* |  | 0 | 0 | 0 | 0 | 0 | 0 | 0 | 0 | 0 | 0 | 0 | 1 | 0 | 0 | 0 | 0 | 0 | 0 | 1 | 507 | 0 | 0 | 0 | 0 | 0 | 0 | 0 | 0 | 0 | 0 | 0 | 0 | 0 | 0 | 0 | 0 | 0 |
|  | Lepidoptera | Noctuidae | *Autographa* |  | 0 | 0 | 0 | 0 | 0 | 0 | 0 | 0 | 0 | 0 | 0 | 0 | 0 | 0 | 0 | 0 | 0 | 9 | 0 | 0 | 0 | 0 | 0 | 0 | 0 | 0 | 0 | 0 | 0 | 0 | 0 | 0 | 0 | 0 | 0 | 0 | 0 |
|  | Lepidoptera | Noctuidae | *Mamestra* | *M. brassicae* | 0 | 0 | 0 | 0 | 0 | 0 | 0 | 0 | 0 | 0 | 0 | 0 | 0 | 0 | 0 | 0 | 0 | 0 | 0 | 6 | 0 | 0 | 0 | 0 | 0 | 0 | 0 | 0 | 0 | 0 | 0 | 0 | 0 | 0 | 0 | 0 | 0 |
|  | Lepidoptera | Pieridae | *Pieris* | *P. rapae* | 0 | 0 | 0 | 1 | 0 | 0 | 0 | 0 | 1 | 10 | 104 | 139 | 0 | 8 | 0 | 0 | 1269 | 185 | 598 | 97 | 0 | 0 | 152 | 0 | 20 | 3 | 3 | 0 | 0 | 0 | 0 | 0 | 1 | 0 | 0 | 0 | 0 |
|  | Lepidoptera | Plutellidae | *Plutella* | *P. xylostella* | 0 | 0 | 0 | 0 | 3 | 2 | 0 | 0 | 0 | 1 | 331 | 0 | 0 | 0 | 0 | 0 | 0 | 1555 | 3 | 35 | 0 | 0 | 0 | 0 | 5 | 6 | 0 | 1 | 0 | 0 | 0 | 0 | 0 | 0 | 0 | 0 | 0 |
|  | Lepidoptera | Pyralidae | *Ephestia* | *E. kuehniella* | 0 | 0 | 0 | 0 | 0 | 0 | 0 | 0 | 0 | 0 | 0 | 0 | 0 | 0 | 0 | 0 | 0 | 0 | 0 | 0 | 0 | 0 | 0 | 0 | 0 | 0 | 9 | 0 | 0 | 0 | 0 | 0 | 0 | 0 | 0 | 0 | 0 |
|  | Mantodea | Mantidae | *Tenodera* | *T. sinensis* | 0 | 0 | 0 | 0 | 0 | 0 | 0 | 0 | 0 | 0 | 0 | 0 | 0 | 0 | 0 | 0 | 0 | 9 | 0 | 0 | 0 | 0 | 0 | 0 | 0 | 0 | 0 | 0 | 0 | 0 | 0 | 0 | 0 | 0 | 0 | 0 | 0 |
|  | Orthoptera | Tetrigidae | *Tetrix* | *T. japonica* | 0 | 2 | 0 | 0 | 0 | 0 | 0 | 0 | 0 | 32 | 0 | 0 | 0 | 0 | 0 | 0 | 0 | 50952 | 198 | 92 | 1 | 0 | 0 | 0 | 0 | 179 | 0 | 0 | 0 | 0 | 0 | 0 | 0 | 3 | 0 | 0 | 0 |
|  | Orthoptera | Tetrigidae | *Tetrix* |  | 0 | 0 | 0 | 0 | 0 | 0 | 0 | 0 | 0 | 0 | 0 | 0 | 0 | 0 | 0 | 0 | 0 | 13 | 0 | 0 | 0 | 0 | 0 | 0 | 0 | 0 | 0 | 0 | 0 | 0 | 0 | 0 | 0 | 0 | 0 | 0 | 0 |
|  | Psocoptera | Liposcelididae | *Liposcelis* |  | 0 | 0 | 0 | 0 | 0 | 2 | 0 | 0 | 3 | 0 | 0 | 30 | 0 | 0 | 0 | 0 | 0 | 0 | 0 | 0 | 0 | 0 | 0 | 0 | 0 | 0 | 0 | 0 | 0 | 0 | 3 | 0 | 0 | 0 | 0 | 0 | 0 |
|  | Psocoptera | Trogiidae | *Cerobasis* | *C. guestfalica* | 0 | 0 | 0 | 0 | 0 | 0 | 0 | 0 | 0 | 0 | 0 | 0 | 1 | 0 | 0 | 0 | 0 | 0 | 0 | 0 | 0 | 0 | 0 | 0 | 0 | 0 | 9 | 0 | 0 | 0 | 0 | 0 | 0 | 0 | 0 | 0 | 0 |
|  | Thysanoptera | Thripidae | *Frankliniella* | *F. occidentalis* | 0 | 0 | 0 | 0 | 0 | 0 | 0 | 0 | 0 | 0 | 0 | 0 | 0 | 0 | 0 | 0 | 0 | 0 | 4 | 0 | 0 | 0 | 0 | 0 | 0 | 0 | 0 | 0 | 0 | 0 | 0 | 0 | 0 | 0 | 0 | 0 | 0 |
|  | Thysanoptera | Thripidae | *Scirtothrips* | *S. dorsalis* | 0 | 0 | 0 | 0 | 0 | 0 | 0 | 0 | 0 | 0 | 0 | 0 | 0 | 0 | 0 | 0 | 0 | 0 | 0 | 0 | 0 | 0 | 0 | 0 | 0 | 0 | 6 | 0 | 0 | 0 | 0 | 0 | 0 | 0 | 0 | 0 | 0 |
|  | Thysanoptera | Thripidae | *Thrips* | *T. tabaci* | 559 | 36 | 136 | 13 | 0 | 2 | 0 | 3 | 2667 | 5 | 488 | 17 | 1323 | 0 | 0 | 0 | 4 | 1040 | 348 | 253 | 1 | 5 | 0 | 0 | 5 | 5 | 568 | 1 | 0 | 0 | 0 | 0 | 23 | 9 | 12 | 91 | 0 |
|  | Thysanoptera | Thripidae |  |  | 0 | 0 | 0 | 0 | 0 | 0 | 0 | 0 | 0 | 0 | 0 | 0 | 0 | 0 | 0 | 0 | 0 | 0 | 0 | 0 | 0 | 0 | 0 | 0 | 0 | 0 | 0 | 0 | 0 | 0 | 0 | 5 | 1 | 0 | 0 | 0 | 0 |

**Table S4**. The list of species detected from surface of cabbage having chewing damage, and their sequence reads.

| Sample  ID | Species | Sequence reads |
| --- | --- | --- |
| S4 | *Thrips tabaci* | 13 |
|  | *Pieris rapae* | 1 |
| S12 | *Pieris rapae* | 139 |
|  | *Diaeretiella rapae* | 1 |
|  | *Thrips tabaci* | 17 |
|  | *Liriomyza sativae* | 17 |
| S21 | *Brevicoryne brassicae* | 3 |
|  | *Thrips tabaci* | 1 |
|  | *Tetrix japonica* | 1 |

**Table S5**. Primers of the first PCR

| **Name** | **1^st^ PCR primers (5´ -> 3´)*** |
| --- | --- |
| mlCOIintF | **ACACTCTTTCCCTACACGACGCTCTTCCGATCTNNNNNN**GGWACWGGWTGAACWGTWTAYCCYCC |
| HCO2198 | **GTGACTGGAGTTCAGACGTGTGCTCTTCCGATCTNNNNNN**TAAACTTCAGGGTGACCAAAAAATCA |

*Bold letters in the sequence indicate sequencing primer and six ambiguous sequences

**Table S6.** Primer for 2^nd^ PCR

|  | **2^nd^ PCR primers (5´ -> 3´)** |
| --- | --- |
| Forward | AATGATACGGCGACCACCGAGATCTACAC**XXXXXXXX**ACACTCTT TCCCTACACGACGCTCTTCCGATCT |
| Riverse | CAAGCAGAAGACGGCATACGAGAT**XXXXXXXX**GTGACTGGAGTT  CAGACGTGTGCTCTTCCGATCT |

*Bolded X indicates the index sequence. A list of index sequences is presented in Table S7
